# The Proteasome Safeguards Red Blood Cell Integrity During Storage and After Transfusion

**DOI:** 10.3390/antiox15091146

**Published:** 2026-09-09

**Authors:** Sandy Peltier, Théo Michel, Fanny Mialane, Mickaël Marin, Michaël Dussiot, Céline Rodriguez, Monika Dzieciatkowska, Marie Tamagne, Camille Roussel, Stéphanie Vicca, Olivier Hermine, Pierre A. Buffet, Benoit Vingert, Steven L. Spitalnik, Angelo D’Alessandro, Michel Prudent, Pascal Amireault

**Affiliations:** 1 Université Paris Cité, INSERM, EFS, BIGR U1134, Team BioTiGR, 75015 Paris, France; sandy.peltier@inserm.fr (S.P.); mickael.marin@inserm.fr (M.M.); camille.roussel@inserm.fr (C.R.); pierre.buffet@inserm.fr (P.A.B.); 2 Université Paris Cité, Institut Imagine, Laboratory of Cellular and Molecular Mechanisms of Hematological Disorders and Therapeutic Implications, INSERM, 75015 Paris, France; michael.dussiot@gmail.com (M.D.); ohermine@gmail.com (O.H.); 3 Initiatives IdEx Globule Rouge d’Excellence (InIdex GR-Ex), Université Paris Cité, 75015 Paris, France; 4 Laboratoire de Recherche sur les Produits Sanguins, Transfusion Interrégionale CRS, 1066 Epalinges, Switzerland; theo.michel@itransfusion.ch (T.M.); michel.prudent@itransfusion.ch (M.P.); 5 Center for Research and Innovation in Clinical Pharmaceutical Sciences, Lausanne University Hospital and University of Lausanne, 1011 Lausanne, Switzerland; 6 Department of Biochemistry and Molecular Genetics, University of Colorado Denver, Anschutz Medical Campus, Aurora, CO 80045, USA; monika.headrick@cuanschutz.edu (M.D.); angelo.dalessandro@cuanschutz.edu (A.D.); 7 EFS Ile de France—Institut Mondor, INSERM, Université Paris-Est Créteil Val de Marne, Eq. Cohen, Groupe Alloréactivité et Aspects Thérapeutiques, 94000 Créteil, France; marie.tamagne@inserm.fr (M.T.); benoit.vingert@inserm.fr (B.V.); 8 Laboratoire d’Hématologie Générale, Hôpital Universitaire Necker Enfants Malades, Assistance Publique–Hôpitaux de Paris (AP-HP), 75015 Paris, France; 9 Laboratoire de Biochimie, Hôpital Universitaire Necker Enfants Malades, Assistance Publique–Hôpitaux de Paris (AP-HP), 75015 Paris, France; stephanie.vicca@aphp.fr; 10 Département d’Hématologie, Hôpital Universitaire Necker Enfants Malades, Assistance Publique–Hôpitaux de Paris (AP-HP), 75015 Paris, France; 11 AP-HP, 75015 Paris, France; 12 Etablissement Français du Sang, Ile-de-France, 94200 Ivry-sur-Seine, France; 13 Department of Pathology and Cell Biology, Columbia University, New York, NY 10032, USA; ss2479@cumc.columbia.edu; 14 Institute of Pharmaceutical Sciences of Western Switzerland, University of Geneva, 1211 Geneva, Switzerland

**Keywords:** red blood cell, erythrocyte, storage, cell aging, cell senescence, transfusion, proteasome, oxidative stress

## Abstract

Pre-transfusion storage of red blood cells (RBCs) induces aging in vitro driven by metabolic and oxidative stress, limiting transfusion efficacy. Unlike nucleated cells where multiple hallmarks characterize aging, proteostasis is expected to play a main role in anucleate RBCs, including proteasomal protein degradation. Although proteasomal activity declines during aging in vitro, its role in generating downstream alterations and post-transfusion clearance remains unclear. We hypothesized that proteasome inhibition accelerates RBC aging in vitro, particularly following re-exposure to physiological conditions. We evaluated the impact of proteasome inhibition (i.e., epoxomicin) on RBC quality during storage and physiological restoration in vitro. Additionally, young and old RBC subpopulations were compared. Proteasome inhibition during storage depleted ATP and altered RBC morphology without immediate oxidative damage. Physiological restoration of proteasome-inhibited RBCs caused accelerated ATP depletion, massive protein aggregation, reduced deformability, hemolysis, and phosphatidylserine exposure, particularly in long-stored RBCs. Strikingly, RBCs aged in vivo also exhibited low proteasomal activity and behaved similarly to stored RBCs following physiological restoration. In conclusion, proteasomal dysfunction is a key hallmark of RBC aging and senescence, driving molecular and cellular modifications that mark RBCs for clearance in vivo. Therefore, enhancing proteasomal function could improve RBC storage quality and transfusion efficacy.

## 1. Introduction

Pre-transfusion refrigerated storage is essential for the ~100 million red blood cell (RBC) concentrates produced worldwide each year [1]. The feasibility of long-term RBC concentrate storage requires the use of storage solutions. Although saline-adenine-glucose-mannitol (SAGM) is currently used in most European countries, alternative additive solutions (i.e., AS-1, AS-3, AS-5, AS-7) are employed in the United States; all aim to preserve RBC quality and sustain metabolism for up to 42 days. However, none are ideal because RBCs continue to age in vitro, undergoing a time-dependent decline collectively known as the “storage lesion” [2].

This phenomenon is characterized by a progressive deterioration of energy metabolism [3,4], marked by declining intracellular ATP levels [5,6,7] and antioxidants [8], alongside increased oxidative stress exposure [9]. Consequently, refrigerated RBCs become increasingly unable to cope with the oxidative stress generated during storage, thereby accelerating lipid and protein oxidation [4,10,11,12,13,14]. These metabolic and oxidative alterations then cause downstream cellular alterations [2], including: increased surface exposure of phosphatidylserine (PS) [6,15], endothelial cell adherence [6], and extracellular vesicle (EV) release [16,17,18]; decreased deformability [19]; morphological changes [16,20]; and ultimately, hemolysis [20]. In Europe, hemolysis is a regulatory quality control metric and must remain below 0.8% at the end of storage [21]. Upon transfusion, these accumulated lesions may induce adverse physiological consequences [2], such as endothelial adhesion [22,23], splenic retention [24,25,26], intra- or extravascular hemolysis [27,28,29], and impaired tissue oxygenation [30,31], thereby reducing transfusion recovery [24,29,32] and efficacy [33,34,35]. The in vitro aging process shares important similarities with physiological aging in vivo, with one main critical distinction: the environment. Thus, in vivo, senescent RBCs circulating in plasma are continuously cleared; in contrast, in vitro, senescent RBCs expressing a clearance-prone phenotype can accumulate within the confined environment of the storage bag, temporarily evading clearance until transfusion.

Long-stored RBCs aged in vitro also share striking similarities with old RBCs physiologically aged in vivo [36,37]. Old circulating RBCs exhibit decreased cell volume [38,39,40], surface area [40], size [41], and deformability [39]. Although metabolic alterations are also evident [37,42], findings regarding ATP levels remain controversial. Indeed, some studies report decreased ATP in old RBCs [38,43,44,45], while others did not observe significant differences [46,47]. Furthermore, old RBCs expose surface PS [38,48], exhibit ionic imbalances [38,45], and are more readily phagocytosed [49].

Mature RBCs are highly differentiated, and their aging process in vivo is most probably distinct from non-circulating nucleated cells. However, advances in understanding nucleated cell aging can certainly inform our understanding of RBC aging. In nucleated cells, the primary hallmarks of aging include genomic instability, telomere shortening, epigenetic alterations, and proteostasis dysfunction [50]. Interestingly, numerous aging-related diseases impacting long-lived highly differentiated neurons, including Alzheimer’s, Parkinson’s, and Huntington’s, are linked to impaired proteostasis [51]. Given that RBCs lack a nucleus and relevant genomic material, most primary nucleic acid-based hallmarks of aging are not applicable. Therefore, proteostasis dysfunction represents the primary aging hallmark that could theoretically be shared by both RBCs and nucleated cells.

Proteostasis is a quality control network essential for maintaining the stability and function of the cellular proteome. In nucleated cells, it comprises four coordinated systems [52,53]: (1) protein synthesis, (2) folding and conformational maintenance by chaperones, (3) protein degradation by the ubiquitin–proteasome system and the autophagy–lysosomal pathway, and (4) spatial sequestration of damaged proteins into aggregates, which are highly toxic to cells. Therefore, proteostasis is particularly critical for RBCs: because these anucleate cells lack de novo protein synthesis capabilities, they cannot replace damaged proteins. Furthermore, they lack lysosomes, rendering the autophagy–lysosomal degradation pathway unavailable. Thus, the entire RBC proteostasis network is limited to protective, reparative, and proteasomal degradative systems [54]. This reliance becomes even more pronounced during refrigerated storage, where cumulative oxidative stress generates a burden of oxidatively damaged proteins [13] that must be efficiently removed to prevent toxicity [53]. Together, these mechanisms repair misfolded proteins or remove altered and oxidized proteins via degradation or sequestration, preventing the accumulation of toxic components.

Although proteasomal activity progressively declines during storage [25,36,55,56,57,58], the causal linkages between this dysfunction and the metabolic impairment, oxidative damage, and cellular alterations leading to post-transfusion RBC clearance remain to be clarified. We previously showed that storage lesions preferentially target a subpopulation of storage-induced microerythrocytes (SMEs) that are rapidly cleared by the recipient’s spleen post-transfusion, identifying them as the senescent RBCs that accumulate during aging in vitro. Notably, SMEs exhibited depleted ATP levels and proteasome activity [25], suggesting that proteostasis dysfunction may constitute a third key molecular impairment underlying the storage lesion, alongside metabolic and oxidative alterations, limiting storage duration. Therefore, we hypothesized that proteasome inhibition in vitro would accelerate storage-related RBC aging and senescence, particularly when the stored RBCs were then exposed to the post-transfusion environment. Thus, our aim was to evaluate the impact of a proteasome inhibitor on RBC quality during storage in vitro at 4 °C, and also in conditions mimicking post-transfusion conditions (i.e., “physiological restoration”).

To this end, we conducted a multiparametric characterization of proteasome-inhibited RBCs during storage (days 7, 28, and 42) and in a model of post-storage physiological restoration in vitro. Molecular phenotypes were identified by analyzing both classical storage lesion markers (e.g., ATP, redox-proteomics) and specific proteostasis markers (e.g., protein-enriched regions, protein aggregates, Heinz bodies). Cellular characteristics were assessed using multiple assays, including morphology, deformability, PS exposure, EV quantification, and hemolysis. Additionally, density-gradient separation provided preparations enriched in young and old RBC subpopulations, allowing comparative analyses of their properties following physiological restoration in vitro.

## 2. Materials and Methods

### 2.1. Experimental Design

#### 2.1.1. Proteasome Inhibition

**Figure 1:**
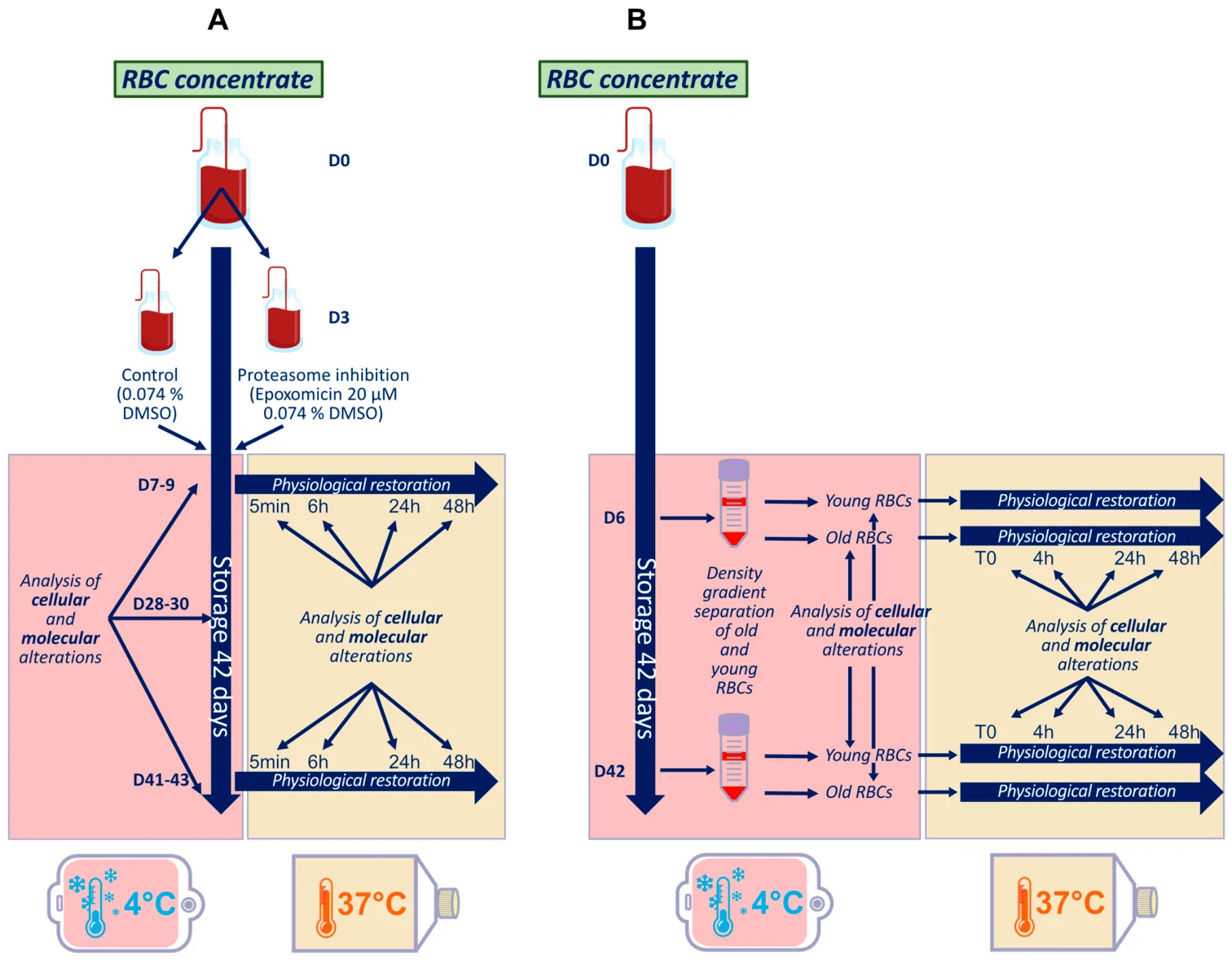
Experimental design. (**A**) RBC concentrates were divided into two small bags: one treated with DMSO (vehicle control) and one with epoxomicin (proteasome inhibitor). Bags were stored in SAGM at 2–6 °C until day 43. Samples were analyzed on storage days 7–9 (short-stored), 28–30 (medium-stored), and 41–43 (long-stored). Short- and long-stored RBCs were exposed to a physiological restoration model in vitro and analyzed after 5 min and 6, 24, and 48 h. Figures 2–6 and supplemental Figures S1–S8 were generated from this experiment. (**B**) Four RBC concentrates were stored in SAGM at 2–6 °C until day 42. At days 6 and 42, RBC samples were collected and separated into two subpopulations: young and old. These subpopulations were analyzed before and after 0 min, and 4, 24, and 48 h of physiological restoration in vitro. Figure 7 and Supplemental Figure S9 were generated from this experiment.

RBC concentrates from healthy donors were obtained from the Etablissement Français du Sang on day 3 of storage. The main characteristics of the RBC concentrates used (i.e., blood type and hematocrit) are provided in Supplemental Table S1. Concentrates were divided into two small bags (#VSE0000A, Macopharma; 25 mL per bag). One bag was treated with 0.074% Dimethylsulfoxide (DMSO, vehicle control) and the second with 20 µM epoxomicin (a potent, irreversible, and specific proteasome inhibitor), in 0.074% DMSO. Bags were stored in SAGM at 2–6 °C until day 43. Samples were aseptically collected and analyzed on storage days 7–9 (short-stored), 28–30 (medium-stored), and 41–43 (long-stored); short- and long-stored RBCs were also analyzed in the physiological restoration model (Figure 1A).

#### 2.1.2. Comparison of Young and Old RBCs

RBC concentrates were obtained from Transfusion Interrégionale CRS (Bern, Switzerland) with donors’ consent, and in agreement with local legislation, from 4 O-RhD+ blood type male healthy donors after a whole blood donation (450 mL). Units were prepared according to standard procedures and according to Swiss national recommendations [59]. Briefly, RBC concentrates were prepared using a top-bottom processing kit (CQ32250, Fresenius Kabi, Bad Homburg, Germany), component-filtered, and stored in SAGM at 4 °C. On days 6 and 42, RBC samples were aseptically collected and separated into two subpopulations: young and old. Subpopulations were analyzed before and during physiological restoration (Figure 1B).

### 2.2. Density Fractionation

RBC concentrate samples were withdrawn on days 6 and 42 of storage and adjusted to 50% hematocrit with 0.9% NaCl solution (freeflex^®^, Fresenius Kabi, Etobicoke, ON, Canada). RBC populations were separated using a Percoll density gradient (ρ = 1.092 g/mL). The 50% RBC solution was carefully layered onto the Percoll solution and centrifuged at 4700× *g* (Rotanta 460R, Hettich, Germany) for 15 min at room temperature. Two fractions were obtained: the top fraction enriched in young RBCs and the bottom fraction enriched in old RBCs. Both fractions were collected separately and washed twice with 0.9% NaCl at 2400× *g* for 15 min at 4 °C. In parallel, a non-fractionated sample was prepared and defined as the “total fraction”. For the sake of readability, density-separated fractions are sometimes referred to as “young” or “old” RBCs. It should be understood that these are subpopulations enriched in young or old cells, and are not pure populations.

### 2.3. Proteasome Activity

For proteasome inhibition experiments, proteasome activity was measured at days 6–8 (i.e., one day prior to functional assays) to confirm proteasome inhibition before proceeding with downstream experiments. For comparison of young and old RBCs, cells were snap-frozen at each time point and stored at −80 °C until further analyses.

The Cell-Based Proteasome-Glo^TM^ Assays kit (Promega, Madison, WI, USA) was used according to the manufacturer’s recommendations, as previously described for RBCs [25]. Briefly, 100,000 RBCs in triplicate were mixed with specific substrates for each activity (i.e., Suc-LLVY, Z-LRR, and Z-nLPnLD for chymotrypsin-like, trypsin-like, and caspase-like activities, respectively), diluted in detection reagent, and incubated for 10 min at room temperature. Luminescence was measured using an Infinite 200 Pro (Tecan, Männedorf, Switzerland) or SpectraMax M3 (Molecular Devices, San Jose, CA, USA).

### 2.4. Physiological Restoration

“Physiological restoration” refers to an in vitro model simulating physiologic conditions in vivo to model the post-transfusion fate of RBCs. It was performed independently by two different laboratories for the two experiments, with minor variations detailed below.

#### 2.4.1. Comparing DMSO and Epoxomicin-Treated RBCs

Physiological restoration was performed with short-stored (days 7–9) and long-stored (days 41–44) RBCs to model the impact of storage duration on their post-transfusion properties. RBCs were diluted to a 5% hematocrit in heat-inactivated fresh frozen AB-RhD+ plasma. Plasma units from 4 donors were obtained from the Établissement Français du Sang, thawed, aseptically pooled together, aliquoted to prevent repeated freeze–thaw cycles, and stored at −20 °C until use. Cells were incubated at 37 °C for 48 h under gentle agitation, as described [60]. The irreversibility of epoxomicin’s effects on proteasome function allowed for continuous proteasome inhibition in this model, even though it was not directly added to the plasma. Samples were collected at 5 min and at 4, 6, 24, and 48 h for subsequent analysis.

#### 2.4.2. Comparing Young and Old RBCs

Following Längst et al. [60], RBC fractions were resuspended in thawed, pooled, fresh frozen plasma (AB-RhD+ group, pooled from 2 male and 2 female donors) at a 10% hematocrit in 200 µL final volume and loaded onto 96-well plates. Plates were incubated (CO_2_ Water Jacketed Incubator Series II, Forma Scientific Inc., USA) under agitation at 37 °C and 5% CO_2_ for 48 h. Samples (pool of 20 wells) were collected after 0, 4, 24, and 48 h of incubation.

### 2.5. Quantifying CFSE^high^ Subpopulations

The CFSE^high^ subpopulation was quantified as described in [25,61]. Briefly, RBCs were stained with 0.05 µM carboxyfluorescein diacetate succinimidyl ester (CFDA-SE) and incubated overnight at 37 °C. The RBC subset with high carboxyfluorescein succinimidyl ester (CFSE) fluorescence intensity (i.e., CFSE^high^ RBCs) was identified by flow cytometry.

### 2.6. Imaging Flow Cytometry

RBCs were resuspended to 1% hematocrit in Krebs-albumax buffer (Krebs–Henseleit buffer [SigmaAldrich, Darmstadt, Germany] modified with 2 g/L glucose, 2.1 g/L sodium bicarbonate, 0.175 g/L calcium chloride dihydrate, and 5g/L lipid-rich bovine serum albumin [Albu-MAX II, Thermo Fisher Scientific, Waltham, MA, USA]). The pH was adjusted to 7.4 immediately prior to use. Samples were analyzed by Imaging flow cytometry (ImageStream X Mark II; Luminex Corporation, Austin, TX, USA). Krebs-albumax buffer, a readily available medium, allows observation of a stable RBC morphology for up to 4 h, similar to that obtained following incubation in plasma [16]. Brightfield images (×60 magnification) were analyzed using dedicated software (IDEAS, version 6.2; Luminex Corporation, Austin, TX, USA) to determine the proportions of SMEs [16].

### 2.7. Mean Corpuscular Volume

Mean corpuscular volume (MCV) was measured with a Sysmex blood analyzer (Sysmex XP-300, Sysmex Corporation, Kobe, Japan).

### 2.8. Post-Translational Modifications and Redox-Proteomics

Proteomics was used to analyze post-translational modifications (PTMs) and redox modifications of RBC proteins. The analyzed PTM was the Gly-Gly residue remnant from ubiquitination. Redox modifications were either reversible (i.e., cysteine mono-oxidation, cysteine glutathionylation) or irreversible (i.e., cysteine di-oxidation, cysteine converted to dehydroalanine) oxidations.

#### 2.8.1. Sample Processing

Ten million RBCs were washed twice in PBS and stored at −80 °C as dry pellets until shipment to the proteomics facility. RBCs were then lysed at a concentration of 4 × 10^6^ cells/mL in methanol:acetonitrile:water (5:3:2, v/v/v). After vortexing at 4 °C for 30 min, lysates were centrifuged for 10 min at 18,000× *g* at 4 °C to pellet proteins, which were stored at −80 °C until analysis. To minimize artifactual oxidation of cysteine thiols during sample preparation, samples were kept cold during processing and handled promptly to minimize exposure to conditions that could promote oxidation. Following preparation, protein lysates were aliquoted and stored at −80 °C, and repeated freeze–thaw cycles were avoided to minimize further oxidation or degradation of thiol-containing proteins.

#### 2.8.2. UHPLC-MS/MS Proteomics

Protein pellets were solubilized in sodium dodecyl sulfate (SDS) and alkylated with 10 mM N-ethylmaleimide (NEM) for 20 min to shield potentially reactive free thiol groups. Following tryptic digestion and peptide cleanup using Filter Aided Sample Preparation (FASP) digestion, the resulting peptides were separated using nano-ultra-high-performance liquid chromatography (UHPLC, nanoElute system) and analyzed on a mass spectrometer (TIMS TOF Pro 2 Single Cell Proteomics, Bruker Daltonics, Bremen, Germany) operated in PASEF mode [62]. The mass spectrometry proteomics data were deposited in the ProteomeXchange Consortium via the MassIVE partner repository with the dataset identifier MSV000102283.

#### 2.8.3. Statistical Analyses for Proteomics

Data processing evaluated both site-specific and global PTMs. For site-specific analysis, only modified peptides detected in at least six out of eight biological replicates under at least one experimental condition were retained for individual quantification. For global analysis, the intensities of all identified modified peptides were summed to calculate the cumulative abundance for each PTM type. Statistical comparisons between control and epoxomicin-treated conditions used two-tailed paired Student’s t-tests. To address multiple testing, we applied a temporal consistency filter; that is, only PTMs exhibiting statistically significant differences (*p* < 0.05) at a minimum of two distinct time points were selected for downstream biological interpretation.

### 2.9. Phosphatidylserine (PS) Exposure

The proportion of RBCs exposing PS was quantified using FITC-conjugated bovine lactadherin (Cat# 9BLAC-FITC, Cryopep, Montpellier, France), as described [6].

### 2.10. Protein-Enriched Regions

We developed a protocol to identify protein-enriched regions using CellTrace^TM^ Yellow (CTY, Thermo Fisher Scientific, Waltham, MA, USA), a fluorescent probe that stains protein amine groups. Briefly, 10 million RBCs were stained with 50 µL of 10 µM CTY for 20 min at room temperature. RBCs were then washed in PBS, centrifuged for 5 min at 450× *g*, and pellets were suspended in 50 µL of Krebs-albumax before acquisition with the ImageStream. Images (×60 magnification) were recorded (INSPIRE software version 200.1.620.0, Luminex Corporation, Austin, TX, USA) in channel Ch03 (560–595 nm), illuminated by the 561 nm laser, and processed using dedicated software (IDEAS, version 6.2, Amnis). Focused single cells were selected using the features gradient RMS_M01_Ch01 and Aspect ratio_M01_Ch01 versus Area_M01_Ch01. RBCs with protein-enriched regions (i.e., CTY spots) were detected using the feature Spot Count_Spot (M03, Channel 3, Bright, 23, 1). Spot size and intensity were calculated using features Area_Spot (M03, Channel 3, Bright, 23, 1) and Intensity_Spot (M03, Channel 3, Bright, 23, 1), respectively.

### 2.11. Protein Aggregates

RBCs containing protein aggregates were detected using the Proteostat^®^ Aggresome detection kit (Enzo Life Sciences, Farmingdale, NY, USA) according to the manufacturer’s recommendations, with some modifications. Due to high variability in staining intensity, test samples were normalized against an internal control.

#### 2.11.1. Preparation of Internal Control

Fresh RBCs (from day 3 RBC concentrate) were biotinylated (25% hematocrit, 10 µL/mL biotin) for 10 min at room temperature. RBCs were then washed twice in PBS and centrifuged for 5 min at 450× *g*. Biotinylated RBCs were stained with streptavidin-APC (2 µg/mL), incubated 15 min at room temperature, then washed in PBS. Streptavidin-APC labeled RBCs were fixed with 1% paraformaldehyde/0.025% glutaraldehyde in PBS for 15 min, washed 3 times in PBS, and plated in a sealed 96-well plate at 5 million RBCs per well. Plates were stored at 4 °C for several months without detectable changes in fluorescence intensity.

#### 2.11.2. Staining

Test RBCs were fixed according to the same protocol as the internal control and stored at 4 °C until analysis. Fixed test RBCs were mixed with one well of internal control, then permeabilized with 0.1% octyl-β-D-glucopyranoside for 15 min at room temperature. RBCs were washed and stained with 200 µL of detection reagent (diluted at 1:500 in 1X kit buffer) for 30 min.

#### 2.11.3. Acquisition and Analysis

Stained RBCs were analyzed by ImageStream without washing. Images (×60 magnification) were recorded (INSPIRE software version 200.1.620.0, Luminex Corporation, Austin, TX, USA) in channel Ch04 (595–642 nm) illuminated by the 488 nm laser (for proteostat spot detection) and in channel Ch11 (642–745 nm) illuminated by the 642 nm laser (for internal control detection), then processed using IDEAS (version 6.2, amnis). Focused single cells were selected using Gradient RMS_M01_Ch01 and Aspect ratio_M01_Ch01 versus Area_M01_Ch01. RBCs with proteostat spots were detected using the feature Max Pixel_Object_4-Proteostat/Mean Pixel_Object_4-Proteostat. The detection threshold was set so that the proportion of positive RBCs in the internal control remained constant (10 ± 0.5%). Spot size and intensity were calculated using Area_Spot (M04, 4-Proteostat, Bright, 40, 5, 0) and Intensity_Spot (M04, 4-Proteostat, Bright, 40, 5, 0), respectively. Results for test RBCs were normalized against those of the internal control.

### 2.12. Heinz Bodies

RBCs diluted to 50% hematocrit in plasma were mixed 1:1 (v/v) with ready-to-use brilliant cresyl blue solution (Cat#1.01384, SigmaAldrich, Darmstadt, Germany) and incubated for 24 h at room temperature. Blood smears were prepared and examined by phase-contrast microscopy; 15 images were captured per condition. Images were anonymized, and RBCs containing Heinz bodies were counted by two blinded observers. Conditions showing a discrepancy of >5% between counts were re-evaluated.

### 2.13. Hemolysis

Total and free hemoglobin (Hb) concentrations were quantified using the Harboe method with the Allen correction [63]. Briefly, samples were centrifuged for 10 min at 1500× *g*, and the supernatants were collected and re-centrifuged to ensure complete cell removal. Total hemoglobin was determined in untreated whole samples, whereas free hemoglobin was measured in the cell-free supernatants. Absorbance was measured in a single determination for each sample using a Nanodrop (Thermo Fisher Scientific, Waltham, MA, USA) at 380, 415, and 450 nm. Hemoglobin concentration (g/dL) was determined using the following formula:

$$Hb=\frac{167.2\times A415-83.6\times A380-83.6\times A450}{100}\times dilution\;factor$$

Hemolysis (%) was calculated as the ratio of free to total hemoglobin, corrected for the plasma volume fraction derived from the sample hematocrit (expressed as a percentage):

$$Hemolysis=\frac{Free\;hemoglobin}{Total\;hemoglobin}\times (100-sample\;hematocrit)$$

### 2.14. Extracellular Vesicles

EVs released into the supernatant were detected by flow cytometry. Their size was estimated using microbeads of known size and absolute numbers were quantified, as described [64]. RBCs were centrifuged for 10 min at 3000× *g* at 4 °C and the supernatant was collected and re-centrifuged for 10 min at 13,000× *g* at 4 °C. The supernatant was collected and frozen at −80 °C until analysis. Supernatants (50 µL) were labeled with 2 μL of lactadherin-FITC (Cat# 9BLAC-FITC, Cryopep, Montpellier, France) and 0.5 μL of anti-GPA-AF647 (Cat# FAB1228R, R&D Systems, part of Bio-Techne, Minneapolis, MN, USA) for 30 min at room temperature; 150 μL of 0.1 μm-filtered PBS was then added to stop the staining reaction.

Microbeads of known size (Megamix-plus SSC and Megamix-plus FSC, Biocytex, Marseille, France) were used to adjust the cytometer parameters (Fortessa X-20, BD Bioscences, Franklin Lakes, NJ, USA) and optimize detection of events <1 µm. Samples were diluted to achieve an acquisition rate of <20,000 events/sec (in LOW acquisition rate), then transferred to Trucount^TM^ tubes (BD Biosciences, Franklin Lakes, NJ, USA) prior to acquisition of 10,000 events within the Trucount bead gate. The absolute concentration of EV (No. of EV/µL) in the supernatant was calculated using the following formula:

$$EV\;concentration=\frac{No.of\;EV\times Total\;No.of\;beads}{No.of\;beads\times Total\;volume}\times dilution$$

where No. of EV = number of GPA+/PS+ EV events; No. of beads = number of bead events acquired; and Total No. of beads = total number of beads per tube (manufacturer’s specification).

### 2.15. Deformability

The elongation capacity of RBCs was evaluated by measuring the elongation index (EI) by ektacytometry using a laser-assisted optical rotational cell analyzer (LORRCA MaxSis, RRMechatronics, De Corantijn, The Netherlands). EI was defined as the ratio of the difference between the two axes of the ellipsoidal diffraction pattern and the sum of these two axes. Measurements were performed across a shear stress gradient ranging from 0.3 to 30 Pa. Although data were acquired for the full range, only results at 30 Pa are presented in the main figures as this high shear stress provided optimal discrimination. Nonetheless, the complete dataset for all shear stresses (0.3–30 Pa) is provided in the Supporting Data S1.

### 2.16. ATP

Intracellular ATP concentrations were determined using an ATP assay kit (ATPlite, PerkinElmer, Waltham, MA, USA). Briefly, ATP concentrations were measured in duplicate after RBC dilution and lysis, calculated using a standard curve (μmol/dL) and normalized against the hemoglobin concentration of each sample (μmol/g Hb).

To compare young and old RBCs, cells were snap-frozen at each time point and stored at −80 °C until further analysis. Samples were deproteinized with perchloric acid and centrifuged to remove precipitated proteins. The supernatant was neutralized using a neutralization solution before ATP quantification.

### 2.17. Oxidized Hemoglobin

HPLC using the Variant II system with the β-thalassemia Short Program (Bio-Rad Laboratories, Hercules, CA, USA) was used to detect hemoglobin variants and quantify oxidized hemoglobin (i.e., the P3 peak).

### 2.18. Statistics

Data were analyzed using GraphPad Prism software, version 9.2.0. Two-way ANOVA followed by Dunnett’s or Šídák’s multiple comparison test was performed for each parameter. The assumptions of normality and homogeneity of residual variance were assessed by visual inspection of Q-Q plots and of plots of residuals versus fitted values, respectively. A *p*-value < 0.05 was considered statistically significant. Statistical results were indicated on the graphs with * (or #) *p* < 0.05, ** (or ##) *p* < 0.01, *** (or ###) *p* < 0.001, and **** (or ####) *p* < 0.0001. The symbol # indicates a significant difference between control and epoxomicin-treated groups, and * indicates a significant difference in the control group at a given time point compared to baseline (day 7 during storage, or 5 min in the physiological restoration model).

## 3. Results

### 3.1. Proteasome Inhibition Exacerbates Specific Morphological and Metabolic Lesions During Storage

Ten RBC concentrates were split into two small bags, one treated with epoxomicin (a proteasome inhibitor) and the other with DMSO (as the vehicle control). Bags were stored in SAGM and analyzed at the beginning, middle, and end of storage to characterize their molecular and cellular profiles. To verify the efficacy of proteasome inhibition, the three proteolytic activities were measured on day 6 of storage. Epoxomicin treatment yielded near-complete inhibition of chymotrypsin-like (101.4 ± 0.3%), trypsin-like (101.5 ± 0.5%) and caspase-like (98.8 ± 0.3%) activities (Supplemental Figure S1A). This inhibition was sustained throughout the entire storage duration (Supplemental Figure S1B).

#### 3.1.1. Proteasome Inhibition Induces Decreased ATP Levels During Storage

To characterize the molecular profile of RBCs, we analyzed levels of two known root causes of storage lesion (i.e., ATP for metabolic alterations and redox-proteomics for oxidative alterations) and markers of a hypothesized third cause: proteostasis. Selected proteostasis markers included three assays to identify distinct types of protein aggregates (i.e., immature protein aggregates using CTY, mature protein aggregates using proteostat, and Heinz bodies using brilliant cresyl blue), as well as HPLC analysis to quantify oxidized hemoglobin.

**Figure 2:**
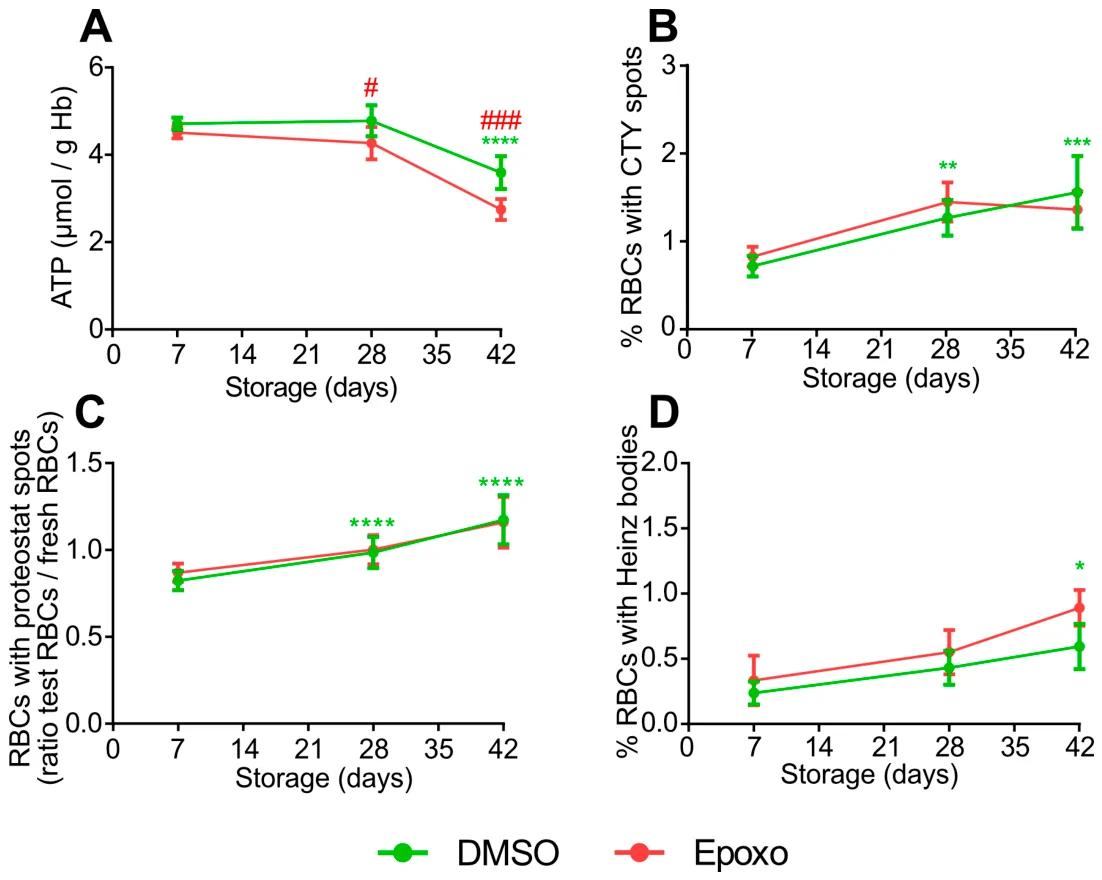
Impact of proteasome inhibition on molecular markers during RBC storage in vitro. RBC concentrates were treated with the proteasome inhibitor, epoxomicin (Epoxo, red circles), or vehicle control (DMSO, green circles), and then stored in SAGM solution at 4 °C for 42 days. Samples were analyzed on days 7, 28, and 42. (**A**) Intracellular ATP levels normalized to hemoglobin content (*n* = 10); (**B**) Percentage of RBCs containing CTY spots (*n* = 8); (**C**) Relative abundance of RBCs containing proteostat spots, expressed as a ratio to fresh RBCs (internal control) (*n* = 8); (**D**) Percentage of RBCs containing Heinz bodies (*n* = 8). Data are presented as mean ± standard error of the mean (SEM). Asterisks indicate significant differences within the DMSO group compared to day 7, determined by two-way ANOVA followed by Dunnett’s multiple comparisons test (* *p* < 0.05, ** *p* < 0.01, *** *p* < 0.001, **** *p* < 0.0001). Hash symbols indicate significant differences between the epoxomicin-treated and DMSO-treated groups at the same time point, determined by two-way ANOVA followed by Šídák’s multiple comparisons test (# *p* < 0.05 ### *p* < 0.001).

Storage in vitro significantly impacted all analyzed parameters. Thus, ATP declined from 4.71 ± 0.14 µmol/g Hb at day 7 to 3.59 ± 0.38 µmol/g Hb at day 42 (*p* < 0.0001, Figure 2A). In addition, a low proportion of RBCs containing CTY spots (1.6 ± 0.4%, *p* < 0.001, Figure 2B) and proteostat spots (1.2 ± 0.1 AU, *p* < 0.0001, Figure 2C) emerged. Heinz bodies were also detected in a minor fraction of RBCs at day 42 (0.6 ± 0.2% RBCs, *p* < 0.05, Figure 2D). Although the oxidized hemoglobin fraction (HPLC-P3) in RBCs slightly decreased during storage (from 5.64 ± 0.08 AU at day 7 to 5.31 ± 0.08 AU at day 42, *p* < 0.0001, Supplemental Figure S2), it concurrently increased in the supernatant (from 8.10 ± 0.81 AU at day 7 to 12.30 ± 0.38 at day 42, *p* < 0.05).

Among these parameters, proteasome inhibition specifically impacted ATP content, with a 23.4% decrease in epoxomicin-treated RBCs (*p* < 0.001, Figure 2A) at day 42. No significant effects were observed on CTY spots (Figure 2B), proteostat spots (Figure 2C), Heinz bodies (Figure 2D), or the HPLC-P3 oxidized hemoglobin fraction (Supplemental Figure S2).

Proteomics analysis quantified ubiquitination and redox-modifications (e.g., cysteine mono-oxidation, di-oxidation, glutathionylation, and conversion to DHA). Heatmap analysis of the five modifications on specific amino acids revealed no major alterations during storage in control RBCs, nor upon epoxomicin treatment, as compared to controls (Supplemental Figure S3A). Global analysis, obtained by summing peptides for each modification, showed an increase in cysteine glutathionylation of non-hemoglobin proteins during storage in control RBCs (Supplemental Figure S3B), without modification upon proteasome inhibition. Other modifications remained stable regardless of storage duration or treatment condition (Supplemental Figure S3B; detailed data are in Supporting Data S1).

These data indicate that, among all molecular parameters evaluated during the refrigerated storage phase, ATP was the only one significantly impacted by proteasome inhibition, showing that proteostasis dysfunction may impact cellular metabolism.

#### 3.1.2. Proteasome Inhibition Exacerbated Morphological Alterations During Storage

Standard cellular parameters associated with the storage lesion were evaluated over 42 days, including morphology, hemolysis, PS exposure, EV production, and deformability.

**Figure 3:**
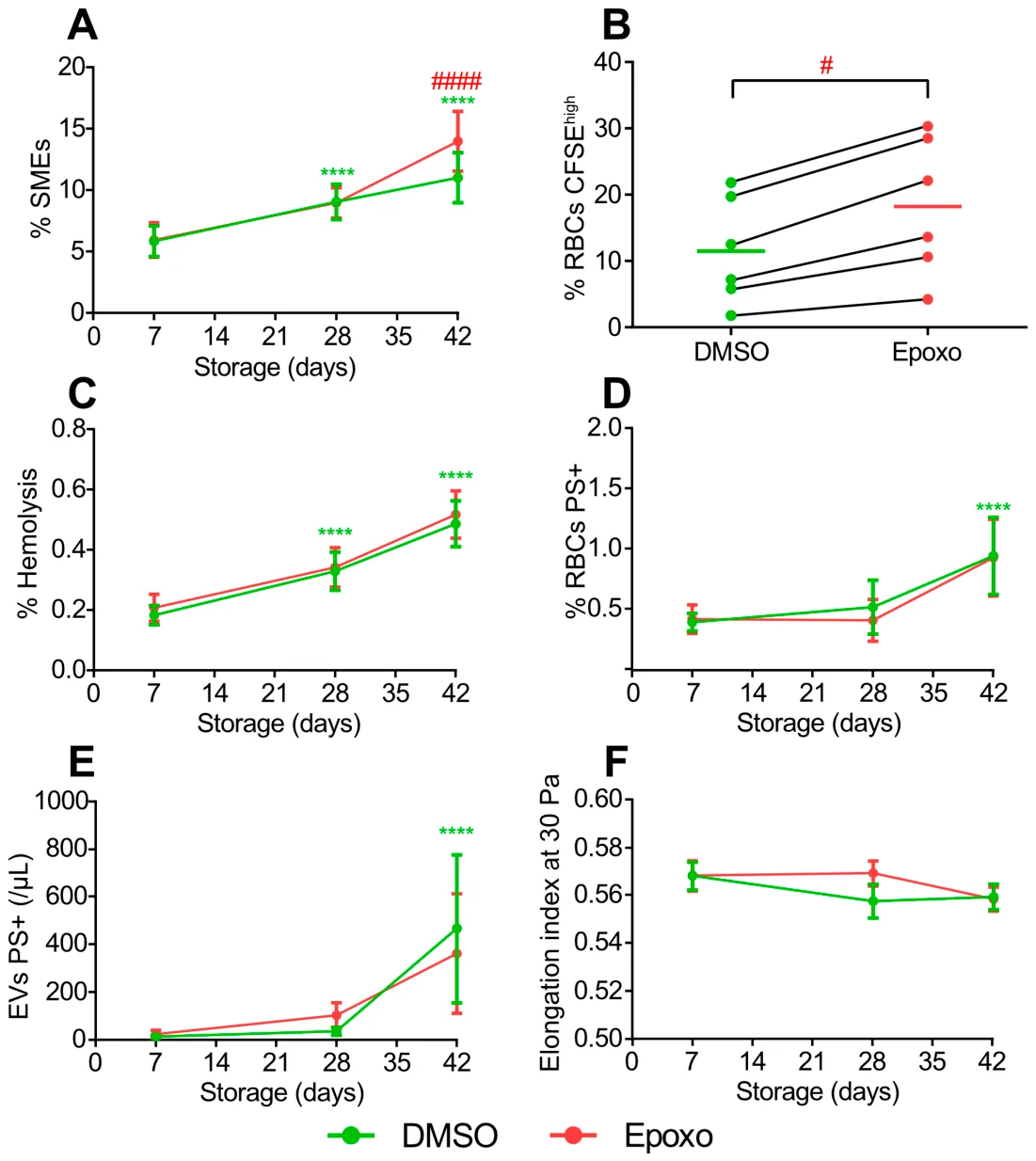
Impact of proteasome inhibition on cellular parameters during RBC storage in vitro. RBC concentrates were treated with the proteasome inhibitor, epoxomicin (Epoxo, red circles), or vehicle control (DMSO, green circles) and then stored in SAGM solution at 4 °C for 42 days. Samples were analyzed on days 7, 28, and 42. (**A**) SME quantification (*n* = 10); (**B**) CFSE^high^ RBCs quantification at day 42 (*n* = 6); (**C**) Hemolysis (*n* = 10); (**D**) Percentage of RBCs exposing PS (*n* = 10); (**E**) EV quantification (*n* = 8); (**F**) Deformability assessed by ektacytometry (*n* = 10). Data are presented as mean ± SEM. Asterisks indicate significant differences within the DMSO group compared to day 7, determined by two-way ANOVA followed by Dunnett’s multiple comparisons test (**** *p* < 0.0001). Hash symbols indicate significant differences between the epoxomicin-treated and DMSO-treated groups at the same time point, determined by two-way ANOVA followed by Šídák’s multiple comparisons test for all panels except (**B**), which was analyzed using the Wilcoxon signed-rank test (# *p* < 0.05, #### *p* < 0.0001).

As expected, storage led to the accumulation of SMEs (11.0 ± 2.0%, *p* < 0.0001, Figure 3A) and CFSE^high^ RBCs (11.5 ± 3.3%, Figure 3B). Storage also triggered hemolysis (0.49 ± 0.08%, *p* < 0.0001, Figure 3C), PS exposure on a minor fraction of RBCs (0.9 ± 0.3% RBCs PS+, *p* < 0.0001, Figure 3D), and EV release (466.4 ± 311.9 EVs PS+/µL, *p* < 0.0001, Figure 3E), while the elongation index at 30 Pa (i.e., deformability) showed a small, but non-significant, decrease (Figure 3F).

Proteasome inhibition exacerbated morphological alterations, including inducing a modest significant increase in SMEs (+3.0%, *p* < 0.0001, Figure 3A) and CFSE^high^ RBCs (+6.8%, *p* < 0.05, Figure 3B). Conversely, other cellular parameters (i.e., hemolysis, PS exposure, vesiculation, and deformability) were unaffected by epoxomicin treatment (Figure 3C–F).

Thus, morphology is the primary cellular parameter affected by proteasome inhibition, suggesting that the observed molecular damage (i.e., proteasome inhibition and ATP depletion) contribute to the spherocytic shift that occurs during storage. ATP and SME levels predict the ability of long-stored RBCs to remain in the circulation post-transfusion [5,24,65,66,67,68,69], suggesting that proteasome-inhibited RBCs would age rapidly in vivo when they are returned to a physiological environment.

### 3.2. Proteasome Inhibition Impairs RBC Fate in a Physiological Restoration Model

An in vitro physiological restoration model was used to characterize the evolution of RBC aging markers in a more physiological environment that mimics post-transfusion conditions in vivo. This involved incubating RBCs in plasma at 37 °C in vitro for up to 48 h. All markers analyzed during storage were re-evaluated after physiological restoration of both short- and long-stored proteasome-inhibited and control RBCs.

#### 3.2.1. Physiological Restoration In Vitro Reveals Altered Metabolism and Proteostasis of Stored Proteasome-Inhibited RBCs, Amplified by Storage Duration

**Figure 4:**
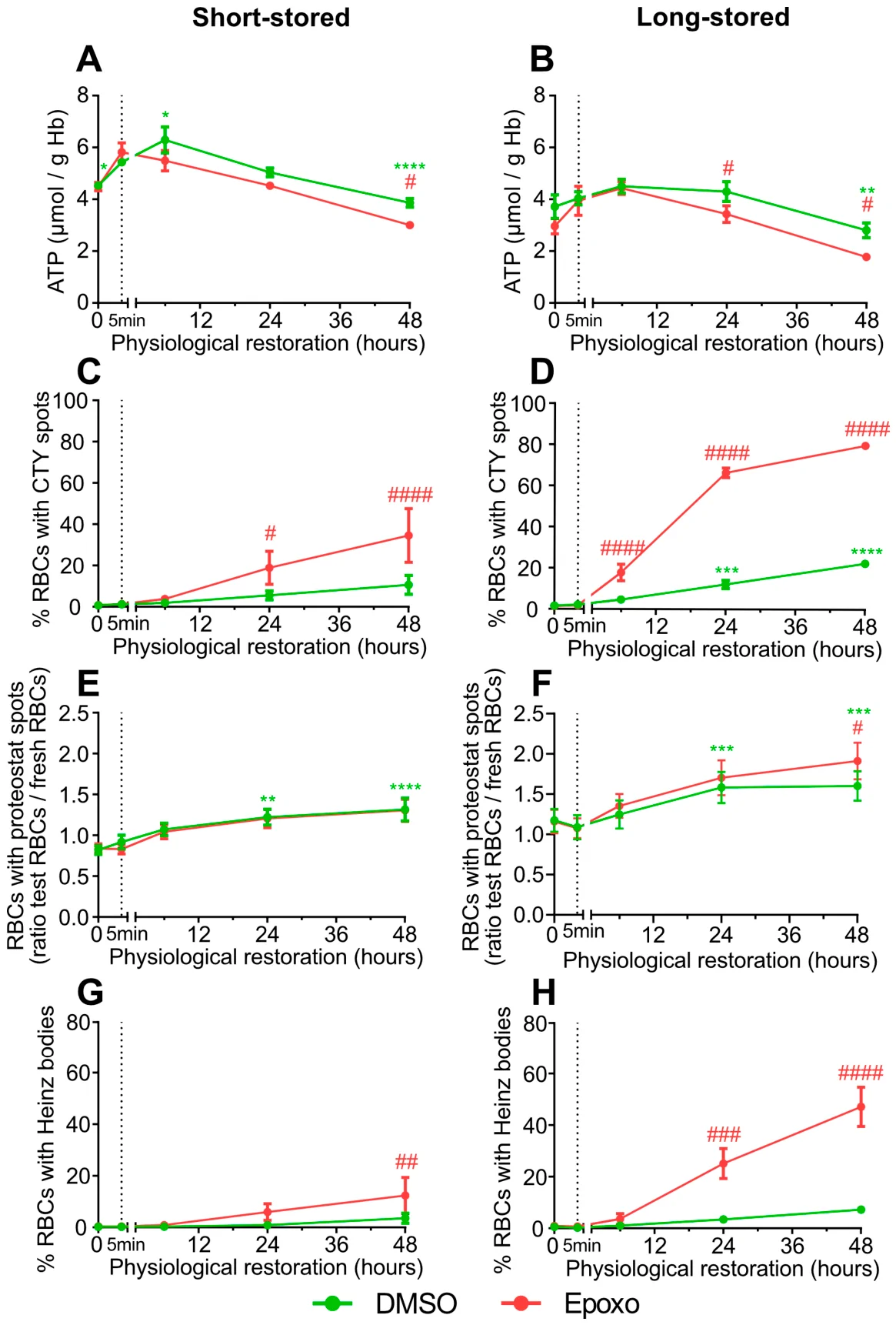
Impact of proteasome inhibition on molecular parameters during RBC physiological restoration. RBC concentrates were treated with the proteasome inhibitor, epoxomicin (Epoxo, red circles), or vehicle control (DMSO, green circles) and then stored in SAGM solution at 4 °C for 42 days. RBCs were exposed to a physiological restoration model on day 7 (short-stored, left panels) and on day 42 (long-stored, right panels). Samples were analyzed before physiological restoration, after 5 min (considered as baseline, vertical dash line), and after 6, 24, and 48 h. (**A**,**B**) Intracellular ATP levels normalized to hemoglobin content (*n* = 7 and *n* = 8); (**C**,**D**) Percentage of RBCs containing CTY spots (*n* = 8); (**E**,**F**) Relative abundance of RBCs containing proteostat spots, expressed as a ratio to fresh RBCs (internal control) (*n* = 8); (**G**,**H**) Percentage of RBCs containing Heinz bodies (*n* = 8). Data are presented as mean ± SEM. Asterisks indicate significant differences within the DMSO group compared to the baseline (5 min), determined by two-way ANOVA followed by Dunnett’s multiple comparisons test (* *p* < 0.05, ** *p* < 0.01, *** *p* < 0.001, **** *p* < 0.0001). Hash symbols indicate significant differences between the epoxomicin-treated and DMSO-treated groups at the same time point, determined by two-way ANOVA followed by Šídák’s multiple comparisons test (# *p* < 0.05, ## *p* < 0.01, ### *p* < 0.001, #### *p* < 0.0001).

Physiological restoration in vitro of control RBCs triggered a biphasic ATP response: an initial rise between 5 min and 6 h, followed by a gradual decline up to 48 h (Figure 4A,B). In long-stored RBCs, this recovery phase restored ATP to levels comparable to those of short-stored RBCs (4.53 ± 0.15 μmol/g Hb in control RBCs at Day 7 T0 vs. 4.48 ± 0.27 μmol/g Hb in control RBCs at day 42 T6h, Figure 4A,B). The proportion of RBCs with CTY spots increased slightly in short-stored controls (10.6 ± 4.6% after 48 h, non-significant, Figure 4C), but rose significantly in long-stored controls from 24 h onward (12.2 ± 2.0% after 24 h, *p* < 0.001 and 22.4 ± 2.0% after 48 h, *p* < 0.0001, Figure 4D). CTY spot size increased during physiological restoration, while CTY spot intensity first increased and then decreased (Supplemental Figure S4). Proteostat spots slightly increased in proportion, size, and intensity regardless of storage duration (Figure 4E,F and Supplemental Figure S5). A very slight and non-significant increase in the proportion of RBCs containing Heinz bodies was also observed (Figure 4G,H). Finally, the oxidized hemoglobin fraction (HPLC-P3) continuously increased during physiological restoration in both short- and long-stored control RBCs (Supplemental Figure S6).

Proteasome inhibition profoundly modified these molecular profiles. In short-stored RBCs, the ATP recovery phase was truncated, with a declining trend as early as 6 h, resulting in significantly lower ATP at 48 h, as compared to controls (3.00 ± 0.14 μmol/g Hb vs. 3.86 ± 0.16 μmol/g Hb, *p* < 0.05, Figure 4A). In long-stored RBCs, although the initial rise was preserved, the subsequent decline was accelerated, leading to critically low ATP levels at 48 h (1.75 ± 0.11 μmol/g Hb vs. 2.78 ± 0.29 μmol/g Hb in control RBCs after 48 h, *p* < 0.05, Figure 4B). Proteasome inhibition induced a significant increase in the proportion of short-stored RBCs with CTY spots at 24 h (+13.4%, *p* < 0.05) and 48 h (+23.9%, *p* < 0.0001, Figure 4C), without changing spot size or intensity (Supplemental Figure S4A,C). In long-stored RBCs, inhibition induced a very strong increase in the proportion of RBCs with CTY spots, beginning as early as 6 h and reaching 80 ± 1% after 48 h of physiological restoration (*p* < 0.0001 compared to control RBCs, Figure 4D), accompanied by a significant increase in spot size (24 h, *p* < 0.001; 48 h, *p* < 0.0001) and intensity (48 h, *p* < 0.001, Supplemental Figure S4B,D). Regarding proteostat spots, inhibition had no effect on short-stored RBCs (Figure 4E and Supplemental Figure S5A,C), but slightly increased the proportion (19% increase, *p* < 0.05, Figure 4F) of spots in long-stored RBCs at 48 h. Nonetheless, spot size and intensity were not significantly affected (Supplemental Figure S5B,D). Proteasome inhibition increased the proportion of RBCs containing Heinz bodies at the beginning of storage (+8.8% after 48 h, *p* < 0.01, Figure 4G), an effect that was accelerated (+21.7% after 24 h, *p* < 0.001) and amplified at the end of storage (+39.9% after 48 h, *p* < 0.0001, Figure 4H and Supplemental Figure S7). Notably, proteasome inhibition induced a slight but significant decrease in the HPLC-P3 oxidized hemoglobin fraction starting from 24 h in short-stored RBCs (6.7 ± 0.09% in proteasome-inhibited RBCs vs. 6.86 ± 0.08% in control RBCs, *p* < 0.001, Supplemental Figure S7).

**Figure 5:**
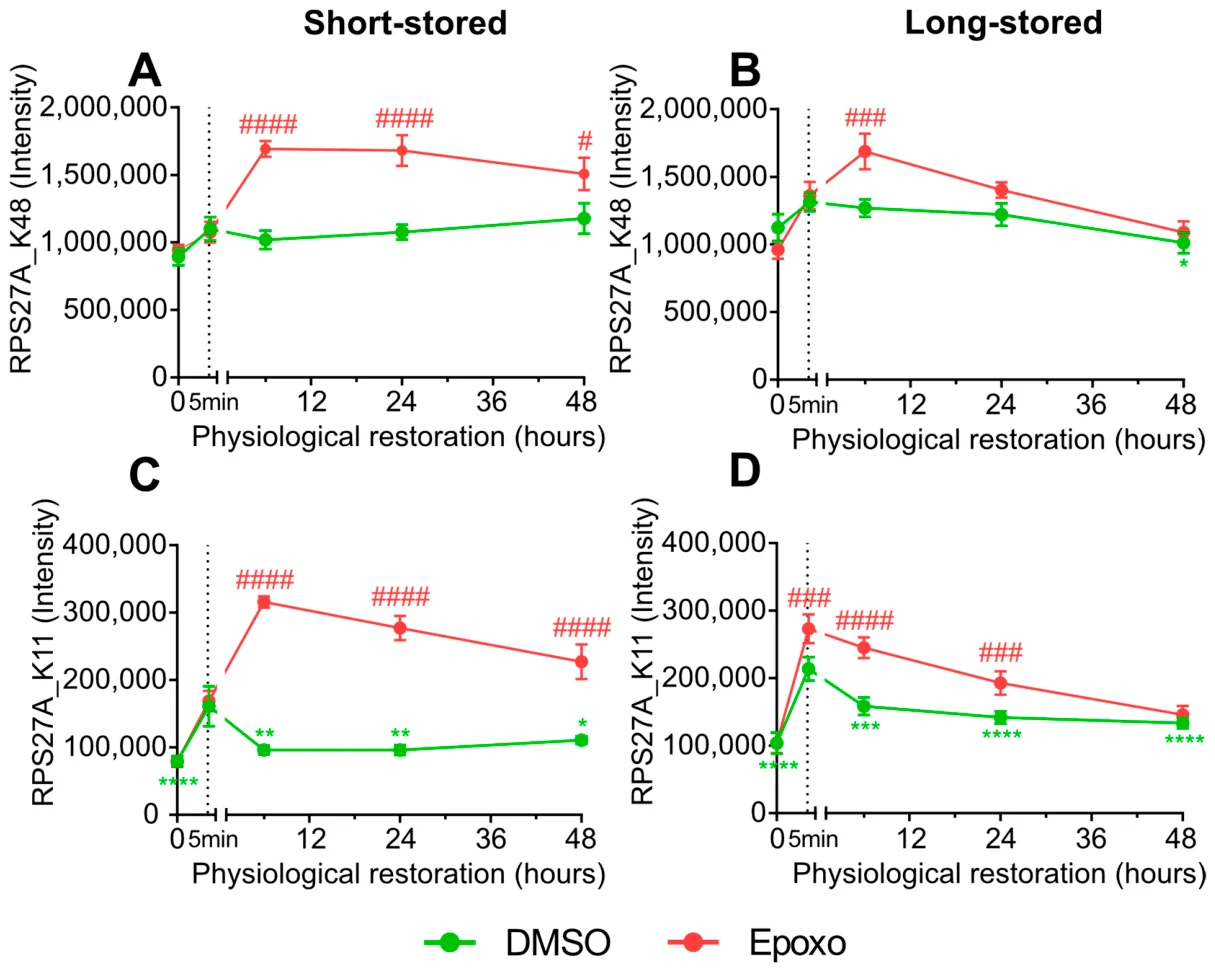
Impact of proteasome inhibition on ubiquitination during physiological restoration of stored RBCs. RBC concentrates were treated with the proteasome inhibitor epoxomicin (Epoxo, red circles) or vehicle control (DMSO, green circles) and stored in SAGM solution at 4 °C for 42 days. RBCs were exposed to a physiological restoration model at day 7 (short-stored, left panels) and day 42 (long-stored, right panels). Samples were analyzed by proteomics before physiological restoration, and after 5 min (considered as baseline, vertical dash line), and 6, 24, and 48 h. (**A**,**B**) Quantification of Gly-Gly residue at Lysine 48 of the RPS27A protein (i.e., ubiquitin) (*n* = 8); (**C**,**D**) Quantification of Gly-Gly residues at Lysine 11 of RPS27A protein (i.e., ubiquitin) (*n* = 8). Data are presented as mean ± SEM. Asterisks indicate significant differences within the DMSO group compared to the baseline (5 min), determined by two-way ANOVA followed by Dunnett’s multiple comparisons test (* *p* < 0.05, ** *p* < 0.01, *** *p* < 0.001, **** *p* < 0.0001). Hash symbols indicate significant differences between the epoxomicin-treated and DMSO-treated groups at the same time point, determined by two-way ANOVA followed by Šídák’s multiple comparisons test (# *p* < 0.05, ### *p* < 0.001, #### *p* < 0.0001).

Analysis of PTMs on specific amino acids during physiological restoration revealed that only one PTM out of the five analyzed types was significantly modified by proteasome inhibition. Specifically, Gly-Gly remnants (a ubiquitination marker) on Lysine 48 (K48) and Lysine 11 (K11) of RPS27A (ubiquitin) were significantly increased upon proteasome inhibition (Figure 5A–D). This accumulation was more pronounced in short-stored than long-stored RBCs. In contrast, global peptide summation indicated that only cysteine mono-oxidation of hemoglobin in short-stored RBCs was modulated by inhibition, showing a decrease after 48 h (*p* < 0.001, Supplemental Figure S8A). All other global PTMs remained unaffected (Supplemental Figure S8B–J). Detailed data are in the Supporting Data S1.

These results demonstrate that RBCs undergo several additional modifications when subjected to the physiological restoration model, revealing fragilities undetected by directly analyzing storage alone. In this model, proteasome inhibition induced molecular metabolic and proteostasis alterations, which were amplified by storage duration. This suggests that active proteostasis protects RBCs from molecular damage when exposed to physiological conditions, especially when RBCs become more fragile due to aging in vitro.

#### 3.2.2. Proteasome Inhibition Exacerbates Cellular Alterations in Long-Stored RBCs

**Figure 6:**
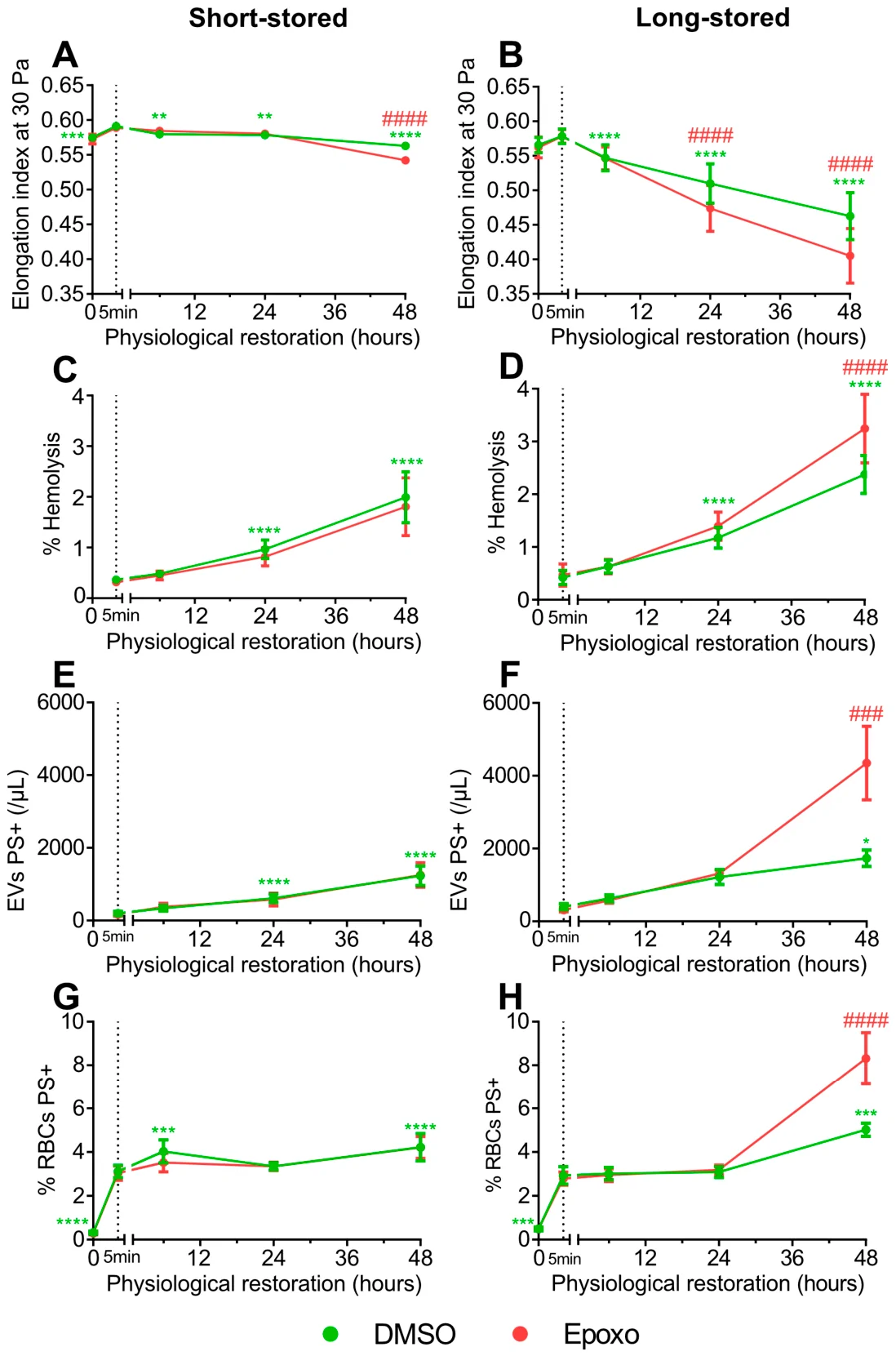
Impact of proteasome inhibition on RBC cellular parameters during physiological restoration. RBC concentrates were treated with the proteasome inhibitor, epoxomicin (Epoxo, red circles), or vehicle control (DMSO, green circles) and then stored in SAGM solution at 4 °C for 42 days. RBCs were exposed to a physiological restoration model on day 7 (short-stored, left panels) and on day 42 (long-stored, right panels). Samples were analyzed before physiological restoration, after 5 min (considered as baseline, vertical dash line), and after 6, 24, and 48 h. (**A**,**B**) Deformability assessed by ektacytometry (*n* = 8); (**C**,**D**) Hemolysis (*n* = 8); (**E**,**F**) EV quantification (*n* = 8); (**G**,**H**) Percentage of RBCs exposing cell-surface PS (*n* = 8). Data are presented as mean ± SEM. Asterisks indicate significant differences within the DMSO group as compared to baseline (5 min), determined by two-way ANOVA followed by Dunnett’s multiple comparisons test (* *p* < 0.05, ** *p* < 0.01, *** *p* < 0.001, **** *p* < 0.0001). Hash symbols indicate significant differences between the epoxomicin-treated and DMSO-treated groups at the same time point, determined by two-way ANOVA followed by Šídák’s multiple comparisons test for all panels except (**B**), which was analyzed using the Wilcoxon signed-rank test (### *p* < 0.001, #### *p* < 0.0001).

The physiological restoration model induced distinct changes in control RBCs depending on their storage duration. In short-stored RBCs, deformability decreased marginally starting at 6 h (1.9% decrease, *p* < 0.01), reaching a 4.7% reduction after 48 h (*p* < 0.0001, Figure 6A). In long-stored RBCs, this decline was steeper (5.4% decrease after 6 h, *p* < 0.0001), reaching a 20.0% decrease after 48 h (*p* < 0.0001, Figure 6B). Hemolysis (Figure 6C,D) and EVs (Figure 6E,F) increased from 24 h onward to a similar extent in short- and long-stored RBCs. PS exposure occurred immediately upon physiological restoration regardless of storage duration (~3% PS+ RBCs at 5 min, Figure 6G,H), with a slight but significant increase after 48 h (4.2% PS+ RBCs, *p* < 0.0001 for short-stored RBCs; 5.0% PS+ RBCs, *p* < 0.001 for long-stored RBCs; Figure 6G,H).

Proteasome inhibition amplified some of these cellular alterations. For example, it exacerbated the loss of deformability in short-stored RBCs after 48 h (3.7% decrease, *p* < 0.0001, Figure 6A), and in long-stored RBCs as early as 24 h (12.4% decrease at 48 h, *p* < 0.0001, Figure 6B). Although hemolysis, EVs, and PS exposure were unaffected in proteasome-inhibited short-stored RBCs (Figure 6C,E,G), they were significantly increased in proteasome-inhibited long-stored RBCs as compared to controls (36.5% increase, *p* < 0.0001, Figure 6D for hemolysis; 151% increase, *p* < 0.001, Figure 6F for EVs; and 66.5% increase, *p* < 0.0001, Figure 6H for PS-exposing RBCs).

Thus, the molecular modifications revealed in the physiological restoration model probably led to cellular alterations. Specifically, proteasome inhibition exacerbated cellular alterations in long-stored RBCs. Therefore, adequate proteostasis is crucial to maintain the cellular integrity of RBCs aged in vitro when they are subsequently exposed to physiological conditions.

### 3.3. Old RBCs Aged In Vivo Have Properties Similar to Those of Proteasome-Inhibited RBCs

**Figure 7:**
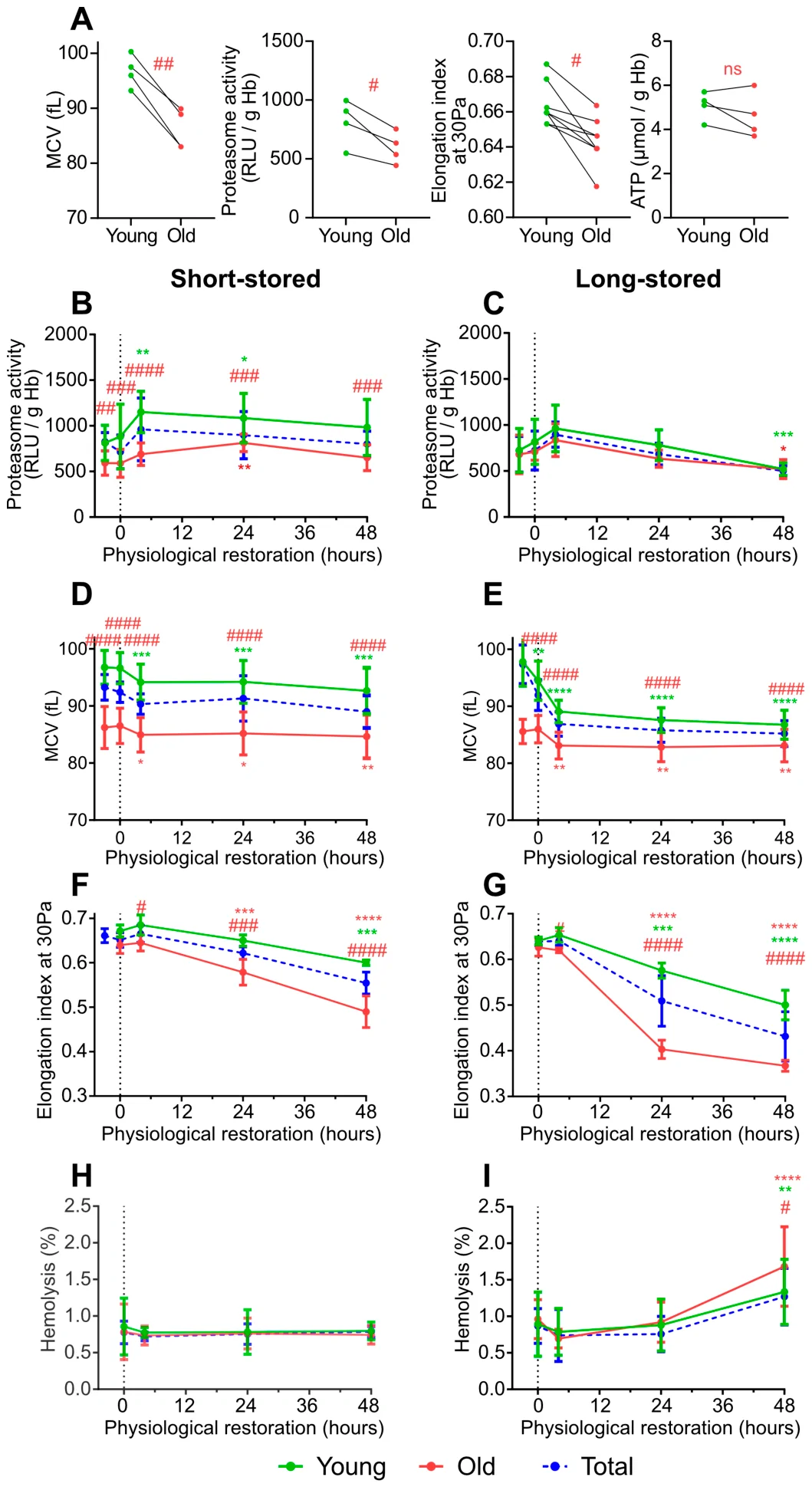
Comparison of young and old RBCs separated from fresh RBC concentrates and during physiological restoration. RBC concentrates were stored in SAGM solution at 4 °C for 42 days. On days 7 and 42, young (green circles) vs. old (red circles) RBC subpopulations were obtained by density fractionation from total RBC concentrates (blue circles). Subpopulations were exposed to a physiological restoration model on day 6 (short-stored, left panels) and on day 42 (long-stored, right panels). Samples were analyzed before physiological restoration, after 0 min (considered as baseline, vertical dash line), and after 4, 24, and 48 h. (**A**) Parameters (e.g., MCV, proteasome activity, deformability, intracellular ATP levels) were compared between young and old RBCs from concentrates stored for 7 days. Data are presented as individual points (*n* = 4). Statistical significance was determined by t-test (# *p* < 0.05, ## *p* < 0.01). (**B**,**C**) Chymotrypsin-like proteasome activity (*n* = 4); (**D**,**E**) MCV (*n* = 4); (**F**,**G**) Deformability assessed by ektacytometry (*n* = 4); (**H**,**I**) Hemolysis (*n* = 4). Data are presented as mean ± SD. Asterisks indicate significant differences within each group (green for young RBCs; red for old RBCs) as compared to the baseline (0 min), determined by two-way ANOVA followed by Dunnett’s multiple comparisons test (* *p* < 0.05, ** *p* < 0.01, *** *p* < 0.001, **** *p* < 0.0001). Hash symbols indicate significant differences between the young and old groups at the same time point, determined by two-way ANOVA followed by Šídák’s multiple comparisons test (# *p* < 0.05, ## *p* < 0.01, ### *p* < 0.001, #### *p* < 0.0001).

To further dissect the role of proteasome activity during RBC aging, we compared properties of young vs. old RBC subpopulations obtained by density fractionation of RBC concentrates. Four RBC concentrates were stored for 42 days; subpopulations were separated at days 6 and 42 and exposed to the physiological restoration model (Figure 7).

In fresh RBC concentrates (day 6), old RBCs exhibited significantly reduced MCV, proteasome activity, and deformability, as compared to young RBCs, whereas ATP levels remained similar (Figure 7A).

During physiological restoration of short-stored RBCs, young RBCs displayed a rapid and transient increase in proteasome activity (*p* < 0.01, 30% increase at T4h, Figure 7B). In contrast, the increase in old RBCs reached significance only after 24 h (*p* < 0.01, 37% increase at T24h). Consistent with their baseline phenotype, old RBCs also showed reduced MCV (Figure 7D) and deformability (Figure 7F) throughout physiological restoration. However, no significant differences were observed in hemolysis (Figure 7H) or ATP levels (Supplementary Figure S9A) between old and young RBCs.

After 42 days of storage, the transient increase in proteasome activity was no longer significant in young or old RBCs upon physiological restoration (Figure 7C). The MCV of young RBCs progressively declined to levels closer to those of old RBCs (Figure 7E). In contrast, old RBCs displayed an exacerbated loss of deformability (Figure 7G), accompanied by increased hemolysis (Figure 7I) and a modest, but significant, reduction in ATP levels (Supplementary Figure S9B) as compared to young RBCs.

Thus, old RBCs aged in vivo possess intrinsically reduced proteasomal activity and cellular alterations that are exacerbated by physiological restoration, mirroring those of proteasome-inhibited RBCs. This confirms that proteostasis is compromised during in vitro and in vivo aging, supporting the hypothesis that its dysfunction is a hallmark of RBC aging.

## 4. Discussion

In transfusion medicine, the premature post-transfusion clearance of a subpopulation of stored RBCs from the recipient’s circulation significantly limits transfusion efficacy [24,29,32,35,70,71]. Therefore, it is critically important to elucidate the mechanisms of RBC aging in vitro during cold storage that precipitate this post-transfusion clearance. Our study demonstrates that, although proteasome inhibition during storage itself did not immediately trigger oxidative or proteostasis stress markers, it did induce a marked energy imbalance characterized by ATP depletion and morphological alterations (Table 1). It is well-established that these two parameters correlate with poor transfusion recovery [5,24,65,66,67,68,69], and aligns with recent findings that morphologically abnormal RBCs are severely ATP-depleted [25]. Although the immediate detrimental effects observed during cold storage were restricted to these markers, we found that inhibiting RBC proteasomes in vitro likely compromises their post-transfusion survival in vivo. This hypothesis is strongly supported by the in vitro physiological restoration model, wherein proteasome-inhibited, long-stored RBCs exhibited extensive molecular (i.e., metabolic, proteostatic) and cellular (e.g., deformability, hemolysis, PS exposure, vesiculation) alterations consistent with rapid clearance in vivo (Table 1).

These results underscore the necessity of evaluating RBCs in vitro not only under static storage conditions, but also under more physiological conditions. Physiological restoration of proteasome-inhibited RBCs uncovered molecular alterations early in storage, even though the RBC profile was completely normal when analyzed directly from the storage bag. Thus, in long-stored RBCs, although proteasome inhibition affected only ATP levels and morphology at 4 °C, physiological restoration at 37 °C revealed severe molecular and cellular damage, ultimately inducing a senescent clearance-prone phenotype (Table 1). Therefore, the physiological restoration model enables detection of latent defects that more accurately predict circulatory RBC fate than standard storage metrics, as previously reported [23,60,72,73,74,75,76].

Strikingly, proteasome-inhibited long-stored RBCs aged in vitro showed similarities with old RBCs aged in vivo. At baseline, old RBCs exhibited reduced cell volume and deformability, with near-normal ATP levels, consistent with the literature [38,39,40,46,47]. Notably, proteasomal activity was reduced in these old RBCs, which, to our knowledge, is reported here for the first time (Table 2). Throughout storage, the gap in cell volume and proteasomal activity between young and old subpopulations narrowed, likely reflecting progressive deterioration of young RBCs until they reached an aging phenotype comparable to pre-storage old RBCs, i.e., aged in vivo before donation (Table 2). Interestingly, similar to proteasome-inhibited RBCs, physiological restoration of old RBCs with intrinsically low proteasome activity exacerbated their defects, triggering additional damage (e.g., ATP depletion, increased hemolysis; Table 2). Thus, proteasome dysfunction due to aging in vivo compromises RBCs’ capacity to manage proteotoxic stress, predisposing them to heightened vulnerability both to storage in vitro at 4 °C and to physiological conditions in vivo at 37 °C. Although old RBCs may initially tolerate physiological restoration moderately well due to sufficient residual proteasomal activity, the progressive accumulation of molecular and cellular damage during aging in vitro may overwhelm their proteasomal capacity, contributing to a vicious cycle accelerating proteasome dysfunction, thereby inducing a senescent clearance-prone phenotype. We propose that the older RBCs present at the time of donation continue to age in vitro, ultimately giving rise to the SME population. These findings support a mechanistic hypothesis linking proteostasis dysfunction to the rapid post-transfusion clearance of SMEs [25], thereby contributing to reduced transfusion efficacy. Collectively, these data strongly suggest that proteasomal dysfunction is a key hallmark of RBC aging and senescence. Proteasome dysfunction impairs the ability of RBCs to degrade damaged proteins. The resulting accumulation causes molecular damage that alters cellular functions, ultimately leading to a senescent clearance-prone phenotype tagging them for removal in vivo.

An intriguing observation is that proteasome inhibition during cold storage did not immediately alter standard proteostasis markers (e.g., protein-enriched regions, protein aggregates, Heinz bodies, oxidized hemoglobin fractions). This suggests that proteasomes are minimally active or required during refrigerated storage, consistent with the slowing of enzymatic reactions at 4 °C. However, ATP levels were nonetheless significantly decreased under these conditions. Because direct modulation of glycolytic pathways by proteasomes is unlikely, this ATP drop likely stems from increased energy consumption rather than reduced production. However, because this study did not specifically investigate the multiple energy-consuming pathways, we can only propose speculative hypotheses. For example, one plausible explanation is a futile cycle of ATP consumption by the ubiquitin–proteasome system itself. Indeed, inhibition of the 20S catalytic core does not prevent upstream ATP-dependent steps, such as target protein ubiquitination or unfolding by the 19S regulatory particle [77]. Consequently, energy may be consumed “unnecessarily” to process proteins that then cannot be degraded, thereby creating a metabolic energy leak. Furthermore, most RBC chaperone proteins (e.g., HSP60, HSP70, HSP90) are ATP-dependent [78]. Thus, in the absence of functional proteasomal degradation, these chaperones are likely more heavily engaged to manage the accumulation of misfolded proteins, thereby leading to increased ATP consumption. Finally, other ATP-dependent processes, such as ionic pumps (e.g., Na^+^/K^+^-ATPase, Ca^2+^-ATPase) and the maintenance of phospholipid asymmetry by ATP-dependent flippases and floppases [79], could be dysregulated or overstimulated in response to proteotoxic stress, thereby contributing to the observed ATP depletion.

ATP depletion compromises cytoskeletal integrity, leading to membrane budding and vesiculation [17,80]. Through continuous membrane loss, RBC morphology becomes progressively altered [16] with reduced deformability [26]. Thus, the energy deficit induced by proteasome inhibition alone may explain the morphological alterations (i.e., increased SMEs and CFSE^high^ RBCs) observed in this study. However, one might have expected concurrent differences in EV production or RBC deformability during storage. The absence of such differences suggests that the methods used to evaluate EV production and RBC deformability at the whole-population level may not be sensitive enough to detect modifications deriving from a subpopulation representing only ~5% of the total sample.

By restoring “normal” metabolism [60], the physiological restoration model unmasked otherwise hidden molecular damage. Thus, even at early stages of storage, inhibited RBCs rapidly accumulated Gly-Gly residue remnants from ubiquitination of lysines K11 and K48 on the RPS27A protein (i.e., ubiquitin itself). These increases in K48 and K11 polyubiquitin chains are canonically associated with proteasomal degradation signaling [81,82]. Their accumulation most probably directly derives from proteasomal system congestion. However, we did not identify specific ubiquitinated target proteins, probably due to a lack of sensitivity of the redox proteomics approach, which was limited to 8 donors. Enrichment of ubiquitinated proteins in future experiments could improve sensitivity of the proteomics analysis.

Imaging identified cytoplasmic inclusions: CTY spots and Heinz bodies. By the end of storage, these inclusions had increased in number and appeared earlier, followed by the appearance of proteostat-positive structures. The kinetics of appearance of these inclusions suggest differences in composition and a specific temporal progression. To interpret the nature of these inclusions in RBCs, where no consensus definition currently exists, we propose a hypothesis based on parallels with the Protein Quality Control (PQC) compartments described in nucleated cells. Thus, typically, in nucleated cells, altered proteins tagged by K11/K48 ubiquitin chains are recognized by the proteasome to be degraded. When this system is incapacitated or overwhelmed, proteins may concentrate into liquid–liquid phase-separated bodies, destined for selective autophagy or temporary sequestration at juxta-nuclear or intranuclear quality control compartments [83]. The accumulation and maturation of these structures can trigger the formation of aggresomes (a juxtanuclear cytoplasmic inclusion surrounded by an intermediate filament cage at the microtubule organizing center), which can also be removed by selective autophagy [83]. If aggresome clearance fails, altered proteins may accumulate as peripheral insoluble protein deposits and potentially be expelled via exocytosis [83]. Although RBCs lack the full machinery of nucleated cells to form canonical PQC compartments, we hypothesize that the inclusions observed in RBCs may correspond to functional equivalents. Thus, based on the biochemical properties of the probes used, we propose the following: CTY, which labels amine groups in proteins, may detect earlier, less-structured assemblies, similar to liquid–liquid phase-separated bodies or juxta-nuclear quality control compartments. Proteostat, which typically detects mature, β-sheet-rich aggregates, may be identifying structures analogous to aggresomes or insoluble protein deposits. Brilliant Cresyl Blue, which precipitates aggregated or concentrated proteins (e.g., denatured hemoglobin and hemichromes), may be visualizing any of the PQC stages. Although further investigation is needed to confirm the exact nature and content of these inclusions, our data demonstrate that proteasome inhibition led to spatial sequestration of altered proteins, potentially including oxidized proteins, via aggregation. Crucially, formation of these protein aggregates may not only be a consequence, but also a cause, of a vicious cycle leading to proteasome dysfunction during aging, both in vitro and in vivo. Indeed, in nucleated cells, large protein aggregates cannot be unfolded and translocated into the narrow 20S proteasome channel; instead, they create steric hindrance that physically clogs the proteasome and inhibits its activity [84]. A similar mechanism has been suggested for RBCs [13] and could function as follows: an initial age-related, temperature-driven (or inhibition-induced) drop in proteasomal activity would promote aggregation, then these aggregates would further suppress proteasomal function by physical clogging, thereby further accelerating cellular decline. This mechanism is supported by the impaired proteasome function observed in RBCs from SOD1-deficient mice [85] and the poor degradation of highly denatured and covalently cross-linked hemoglobin molecules in RBC lysates [86].

Paradoxically, in short-stored, proteasome-inhibited RBCs, we observed a decrease both in the HPLC-P3 oxidized hemoglobin fraction and in hemoglobin cysteine oxidation. This probably results from either the sequestration of oxidized proteins into inclusions (thereby rendering them undetectable) or their elimination via vesiculation. This hypothesis could also explain why an increase in oxidized proteins throughout storage was not detected, although other studies report the accumulation of oxidized proteins [4,11,13,14], again suggesting a potential limitation in sensitivity given our experimental design.

The theory of vesiculation as a mechanism to remove oxidized proteins was previously proposed [13,54,87,88,89] and is supported by our observation of increased PS-positive EVs in the supernatant during physiological restoration. Thus, RBCs likely try to eliminate damaged components and cytoplasmic inclusions by vesiculation, leading to morphological alterations. Unfortunately, we were unable to analyze RBC morphology during physiological restoration due to artifactual stomatocytic transformation. This transformation, which reduces the projected surface area, prevents precise morphological analysis. Although this phenomenon was observed previously [60], it is rarely reported, likely because morphology is infrequently analyzed in depth under such conditions. Nonetheless, extensive vesiculation leads to a decreased surface-to-volume ratio and reduced deformability [26]. Collectively, these alterations compromise membrane integrity, ultimately causing lysis of the most severely damaged RBCs and impairing the circulatory ability of the remaining RBC population. Future studies using in vivo models and/or more complex physiological restoration systems will be required to refine these observations. Nevertheless, the current approach successfully reveals alterations that are invisible during cold storage, proving its relevance for challenging RBCs under conditions closer to normal physiology in vivo and providing additional key information regarding their post-transfusion fate.

Our results should be interpreted with respect to several additional limitations. First, the RBC concentrates used for pharmacological proteasome inhibition were of superior quality as compared to those typically observed. This high quality was evidenced by comparing the morphologically altered RBC proportions [16,25,61], ATP levels [6,16], deformability [6,16,19,90,91,92,93], and oxidized proteins [4,10] reported in other studies with the current data. Thus, the exceptional quality of our RBC concentrates likely led to reduced engagement of the proteasomal degradation system, thereby potentially underestimating the impact of proteasome inhibition. This high quality may be attributed to the use of small storage bags, which offer a higher “bag surface area/RBC volume” ratio than standard bags (1.6 cm^2^/mL vs. 0.68–1.02 cm^2^/mL), likely favoring Di(2-ethylhexyl) phthalate (DEHP) incorporation into the RBC membrane, a plasticizer that increases membrane fluidity, thereby providing protection [94]. This suggests that the importance of proteasomal function could be even greater under standard or suboptimal storage conditions (e.g., irradiated RBC concentrates [95,96], G6PD-deficient donors [97]). Additionally, omics experiments were conducted on a limited number of donors with significant inter-donor variability, necessitating rigorous data cleaning, which consequently reduces the depth of analysis. Finally, although the physiological restoration model successfully revealed latent alterations in vitro, it is only a proxy for clearance, rather than a direct demonstration of RBC removal in vivo. Indeed, unlike the dynamic environment in vivo, this in vitro system lacks endothelial interactions, shear stress, fluctuation in gas exchange, and reticuloendothelial clearance mechanisms. In addition, the low hematocrit (i.e., 5–10%) and static incubation conditions differ from physiological blood flow. Consequently, although our data provide valuable insights into the presumed post-transfusion fate of RBCs, future studies in vivo will be required to validate these findings further.

From a clinical perspective, these observations support the hypothesis that proteasome activity is a key determinant of storage quality and post-transfusion viability. Thus, on average, 25–31% of long-stored volunteer donor RBCs are morphologically altered (i.e., SMEs), demonstrate low proteasomal activity, and are rapidly cleared from the circulation post-transfusion [24,25]. Although this represents a mean value, the inter-individual variability in the SME proportions of donated RBCs after 42 days of storage is substantial, ranging from 4.9% to 68.1% [24]. Although reduced transfusion efficacy of individual long-stored RBC units is unlikely to cause serious adverse events in non-life-threatening acute setting (e.g., the transfusion of 2 units following a hip replacement in an otherwise healthy patient), the need to provide more frequent transfusions because some contain a high proportion of rapidly cleared SMEs could contribute to various complications, such as transfusion-related iron overload in chronically transfused patients (such as those with sickle cell disease or myelodysplastic syndrome) [98,99]. Taken together, these findings pave the way for future studies evaluating new RBC preservation approaches. In addition to promising strategies that aim to limit oxidative damage by antioxidant supplementation [100,101,102,103] or hypoxic storage [65,104], or sustain metabolism by pH modulation [105,106,107,108] or carnitine supplementation [109,110], pharmacological activation of the ubiquitin–proteasome pathway during refrigerated storage may be promising for improving RBC storage quality by limiting the proportion of SMEs. By maintaining the ability to eliminate oxidized proteins and prevent formation of steric-hindering aggregates, it may be possible to preserve the ATP pool, limit membrane rigidification, and ultimately improve transfusion recovery, particularly for long-stored RBC concentrates. This strategy could also be applied to extend the lifespan of RBCs in hemoglobinopathies. For example, in β-thalassemia the proteasome is essential for limiting hemoglobin aggregation by degrading excess α-chains [111], which may contribute to premature RBC clearance [112,113,114]. Enhancing proteasomal activity could therefore potentially reduce transfusion needs.

## 5. Conclusions

In conclusion, the current study sheds light on the cascade of events occurring in mature RBCs after proteostasis dysfunction caused by proteasome inhibition; these range from molecular damage, which generates cellular alterations, to the ultimate appearance of a clearance-prone phenotype. Thus, we propose that proteostasis dysfunction may represent a third key molecular alteration, alongside oxidative and metabolic alterations, driving the cellular changes responsible for the RBC storage lesion and leading to reduced transfusion efficacy. This study also highlights the central role of the proteasome in maintaining RBC homeostasis during aging, whether during 4 °C storage in vitro or while circulating in vivo. Proteasomal efficiency determines the ability of RBCs to manage the protein damage that likely results from oxidative stress, which becomes increasingly critical as RBCs age in vivo or undergo prolonged refrigerated storage in vitro. We hypothesize that the accumulation of protein aggregates, acting as both a symptom and a cause of proteasomal clogging, drives a cascade of cellular deterioration culminating in RBC vesiculation and RBC clearance. Future studies should explore whether enhancing proteostasis functioning can improve the storage quality and transfusion efficacy of refrigerator-stored RBC concentrates, as well as slow premature RBC aging in vivo in patients with hemoglobinopathies.

## Figures and Tables

**Table 1:** Impact of proteasome inhibition on RBC concentrates during storage and physiological restoration. The effects of proteasome inhibition are presented by a color gradient indicating the intensity of the impact: shades of pink/brown indicate a negative impact, shades of blue indicate a positive impact, and white indicates no impact. The color intensity correlates with the magnitude of the effect. NA: not applicable.

Analyzed Parameters		Storage	Physiological Restoration	
			Short- Stored	Long-Stored
Molecular properties	ATP			
	Protein-enriched regions			
	Protein aggregates			
	Heinz bodies			
	Ubiquitination			
	Oxidized hemoglobin *			
	Other oxidized proteins			
Cellular properties	Morphology		NA	NA
	Hemolysis			
	EV			
	Deformability			
	PS			

* (HPLC/proteomic).

**Table 2:** Impact of aging on RBCs during aging in vivo and by physiological restoration after aging in vitro. The effects of aging are presented by a color gradient indicating the intensity of the impact: shades of pink/brown indicate a negative impact and white indicates no impact. The color intensity correlates with the magnitude of the effect. NA: not applicable.

Analyzed Parameters		Aging	Physiological Restoration	
			Short- Stored	Long- Stored
Molecular properties	Proteasome			
	ATP			
Cellular properties	MCV			
	Hemolysis	NA		
	Deformability			

## Data Availability

The values for all data points in graphs are reported in the Supporting Data S1. Mass spectrometry proteomics data are openly available in the ProteomeXchange Consortium via the MassIVE partner repository with the dataset identifier MSV000102283.

## Supplementary Materials

The following supporting information can be downloaded at: https://www.mdpi.com/article/10.3390/antiox15091146/s1, Supplemental Table S1: Characteristics of the RBC concentrates used; Supplemental Figure S1: Quantification of proteasome inhibition after epoxomicin treatment; Supplemental Figure S2: Quantification of HPLC-P3 oxidized hemoglobin fraction in RBCs and supernatant during storage in vitro; Supplemental Figure S3: Analysis of post-translational modifications by proteomics during RBC storage in vitro; Supplemental Figure S4: Impact of proteasome inhibition on CTY spots during physiological restoration of stored RBCs; Supplemental Figure S5: Impact of proteasome inhibition on proteostat spots during RBC physiological restoration of stored RBCs; Supplemental Figure S6: Impact of proteasome inhibition on HPLC-P3 oxidized hemoglobin during physiological restoration of stored RBCs; Supplemental Figure S7: Impact of proteasome inhibition on Heinz body production during physiological restoration of stored RBCs; Supplemental Figure S8: Proteomic analysis of post-translational modifications during physiological restoration of stored RBCs; Supplemental Figure S9: Comparison of intracellular ATP level in young and old RBCs during physiological restoration.

## Abbreviations

The following abbreviations are used in this manuscript:

RBC	Red blood cell
CFSE	Carboxyfluorescein succinimidyl ester
CTY	CellTrace Yellow
DMSO	Dimethylsulfoxide
EV	Extracellular vesicle
HPLC	High-Performance Liquid Chromatography
MCV	Mean corpuscular volume
PQC	Protein Quality Control
PS	Phosphatidylserine
PTM	Post-translational modification
SAGM	Saline-adenine-glucose-mannitol
SME	Storage-induced microerythrocyte
