# Supplementary material for: The Proteasome Safeguards Red Blood Cell Integrity During Storage and After Transfusion
Source: Antioxidants (Basel). 2026 Sep 9;15(9):1146. doi: 10.3390/antiox15091146 (PMC13603181; doi:10.3390/antiox15091146)
Supplement: Supplementary file 1 [file antioxidants-15-01146-s001.zip › antioxidants-4446981-supplementary.pdf]

Supplemental Table S1. Characteristics of the RBC concentrates used. Blood type, hematocrit, donor's age and biological sex for each RBC concentrate are provided. N/A : not available.

Donor	Blood Type	Hematocrit (%)
**296**	A-	N/A
**298**	B+	N/A
**299**	O+	N/A
**300**	O+	N/A
**301**	O+	N/A
**325**	O+	67
**326**	O+	54
**337**	O-	55
**338**	A-	55
**339**	O+	54
**340**	A+	50
**341**	O+	50
**342**	A+	50
**343**	A+	51
**344**	O+	54
**A**	O+	61
**B**	O+	61
**C**	O+	61
**D**	O+	56

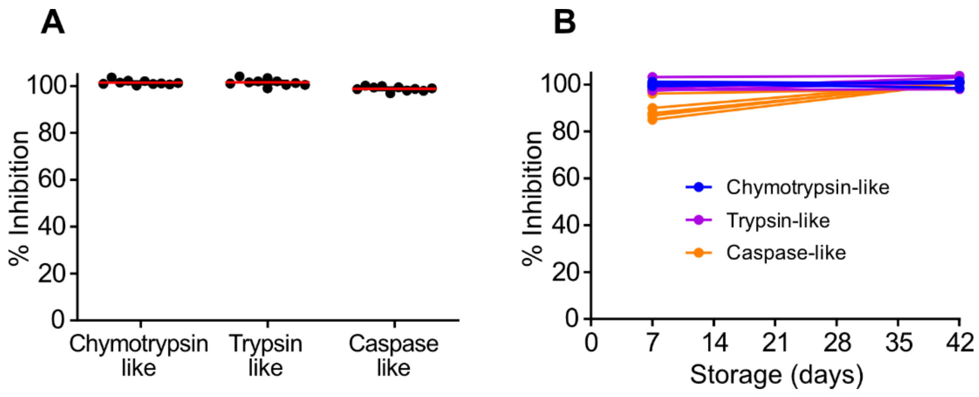

Supplemental Figure S1. Quantification of proteasome inhibition after epoxomicin treatment. RBC concentrates were treated with the proteasome inhibitor epoxomicin or vehicle control (DMSO) and stored in SAGM solution at 4°C for 42 days. (**A**) Proteasome activities of the analyzed RBC concentrates, 3 days after inhibition (*n*=10). (**B**) Proteasome activity of epoxomicin-inhibited RBC concentrates at the beginning and end of storage (*n*=5); these RCCs were not used for subsequent analysis. Data are presented as individual points.

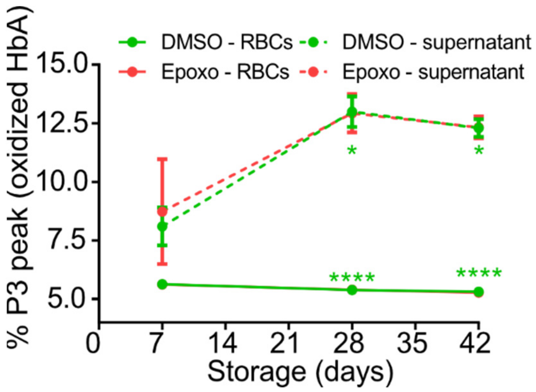

Supplemental Figure S2. Quantification of HPLC-P3 oxidized hemoglobin fraction in RBCs and supernatant during storage in vitro. RBC concentrates were treated with the proteasome inhibitor, epoxomicin (Epoxo, red circles), or vehicle control (DMSO, green circles) and then stored in SAGM solution at 4°C for 42 days. Samples were analyzed on days 7, 28, and 42. HPLC-P3 oxidized hemoglobin was quantified in RBCs (solid lines, *n*=8) and supernatant (dashed lines, *n*=3). Data are presented as mean ± standard error of the mean (SEM). Asterisks indicate significant differences within the DMSO group compared to day 7, determined by two-way ANOVA followed by Dunnett's multiple comparisons test (* $p < 0.05$, **** $p < 0.0001$). Hash symbols indicate significant differences between the epoxomicin-treated and DMSO-treated groups at the same time point, determined by two-way ANOVA followed by Šídák's multiple comparisons test (non-significant).

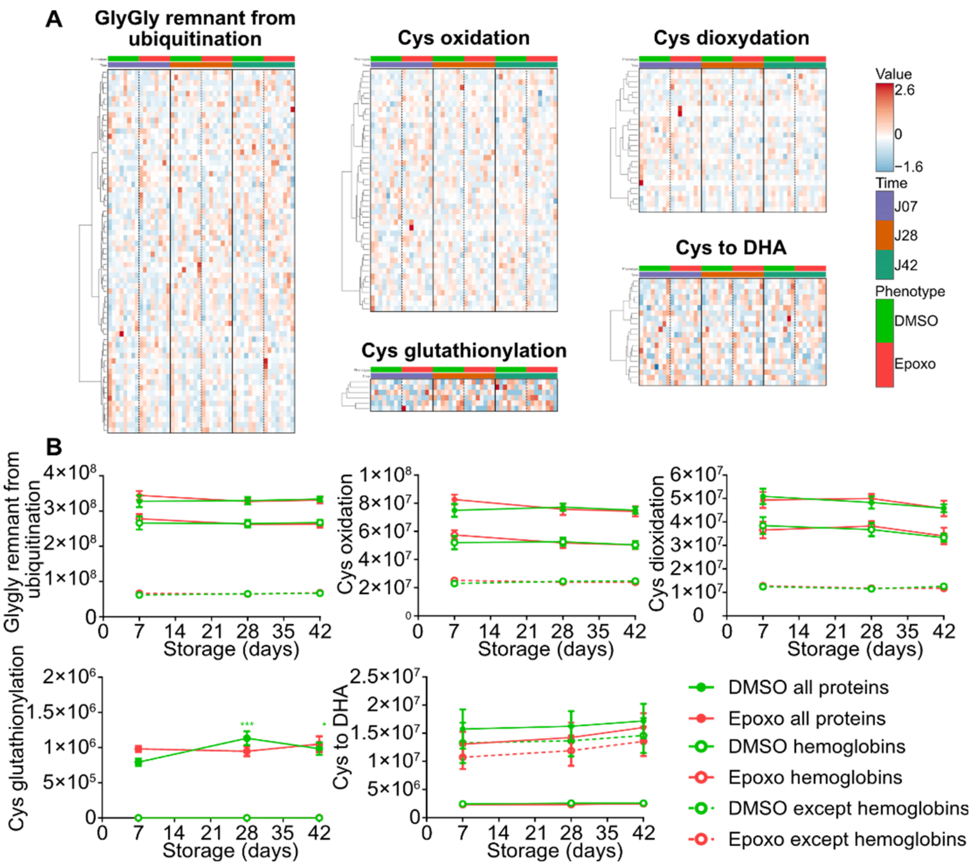

Supplemental Figure S3. Analysis of post-translational modifications (PTMs) by proteomics during RBC storage in vitro. RBC concentrates were treated with the proteasome inhibitor, epoxomicin (Epoxo, red circles), or vehicle control (DMSO, green circles) and then stored in SAGM solution at 4°C for 42 days. Samples were analyzed by proteomics on days 7, 28, and 42. (**A**) Heatmap of Gly-Gly remnants from ubiquitination, cysteine mono-oxidation, cysteine di-oxidation, cysteine glutathionylation, and cysteine converted to DHA. Only modifications detected in at least six out of eight biological replicates under at least one experimental condition were retained (*n*=8). (**B**) Global analysis obtained by summing intensities of all identified modified peptides. Proteins were divided into three groups: all proteins (solid circles with solid lines), hemoglobins (empty circles with solid lines), and all proteins except hemoglobins (empty circles with dashed lines). Data are presented as mean ± SEM (*n*=8). Asterisks indicate significant differences within the DMSO group compared to day 7 (* $p < 0.05$, *** $p < 0.001$). Hash symbols indicate significant differences between the epoxomicin-treated and DMSO-treated groups at the same time point (non-significant).

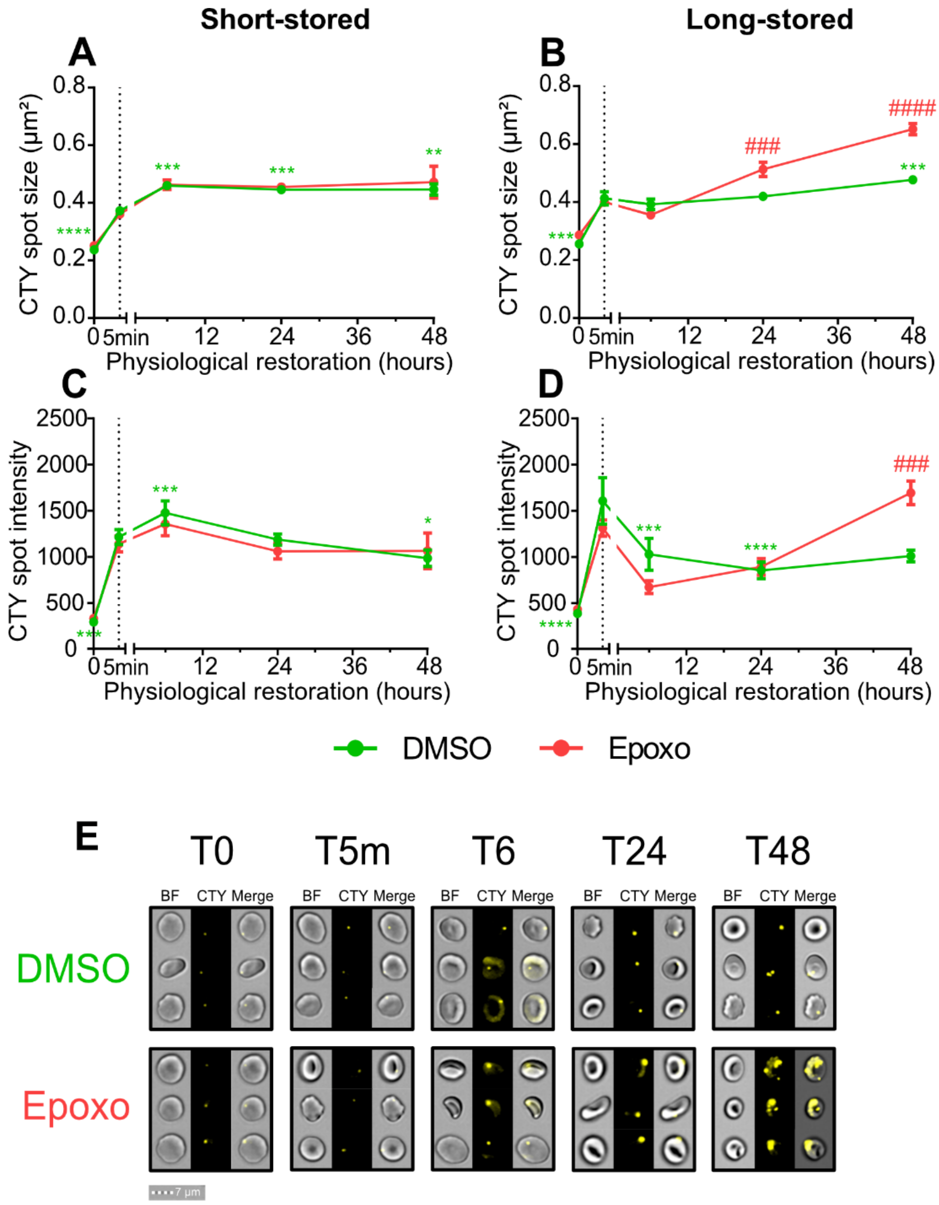

Supplemental Figure S4. Impact of proteasome inhibition on CTY spots during physiological restoration of stored RBCs. RBC concentrates were treated with the proteasome inhibitor, epoxomicin (Epoxo, red circles), or vehicle control (DMSO, green circles) and then stored in SAGM solution at 4°C for 42 days. RBCs were exposed to a physiological restoration model on day 7 (short-stored, left panels) and on day 42 (long-stored, right panels). Samples were analyzed before physiological restoration, and after 5 minutes (considered as baseline, vertical dash line), and 6, 24, and 48 hours. (**A**, **B**) CTY spot size (*n*=8); (**C**, **D**) CTY spot intensity (*n*=8). (**E**) Representative images of long-stored RBCs containing CTY spots (i.e., yellow dots) exposed to the physiological restoration model. Data are presented as mean ± SEM. Asterisks indicate significant differences within the DMSO group compared to the baseline (5 minutes), determined by two-way ANOVA followed by Dunnett's multiple comparisons test (* $p < 0.05$, ** $p < 0.01$, *** $p < 0.001$, **** $p < 0.0001$). Hash symbols indicate significant differences between the epoxomicin-treated and DMSO-treated groups at the same time point, determined by two-way ANOVA followed by Šídák's multiple comparisons test (### $p < 0.001$, #### $p < 0.0001$). BF: Brightfield

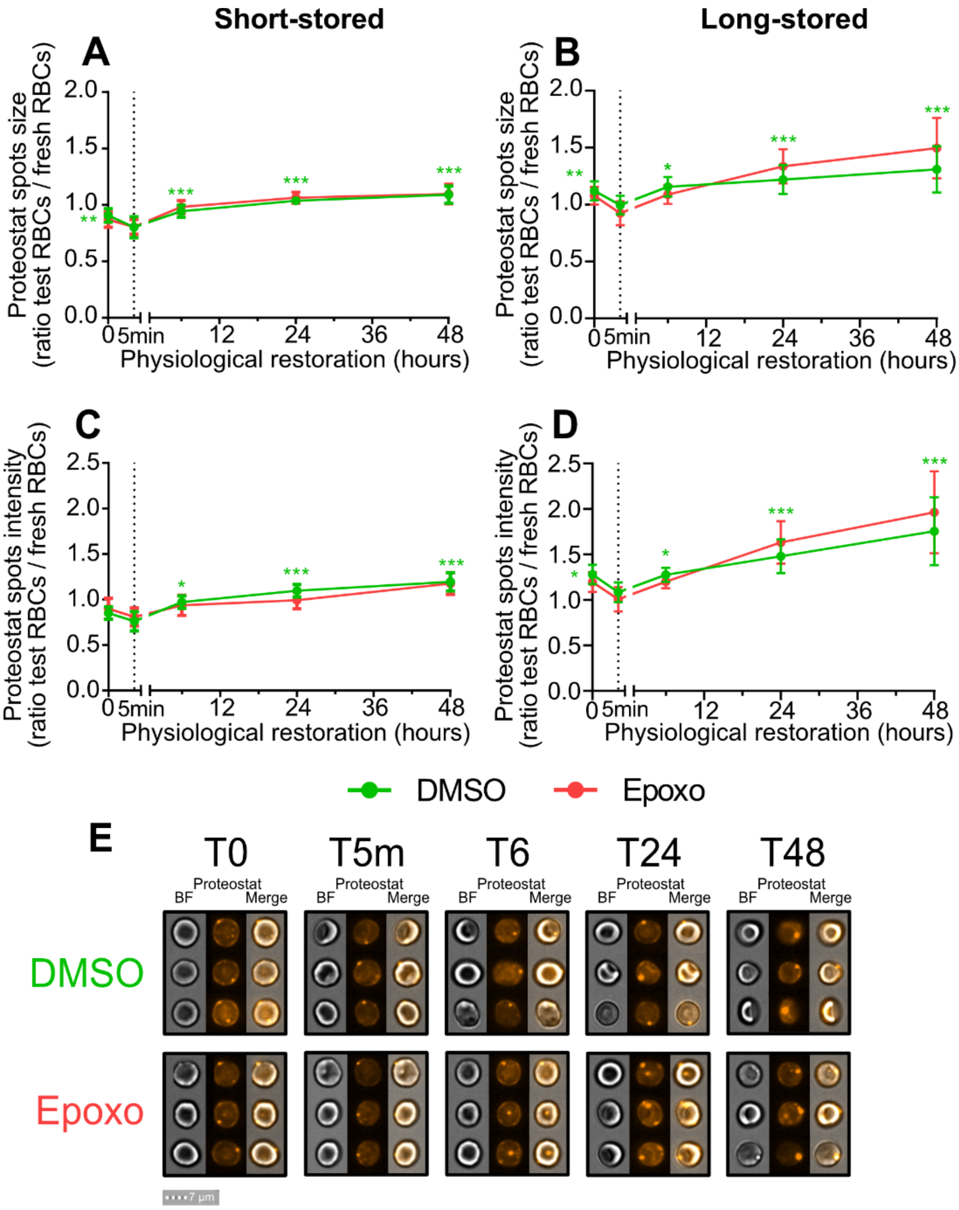

Supplemental Figure S5. Impact of proteasome inhibition on proteostat spots during physiological restoration of stored RBCs. RBC concentrates were treated with the proteasome inhibitor, epoxomicin (Epoxo, red circles), or vehicle control (DMSO, green circles) and then stored in SAGM solution at 4°C for 42 days. RBCs were exposed to a physiological restoration model on day 7 (short-stored, left panels) and on day 42 (long-stored, right panels). Samples were analyzed before physiological restoration, and after 5 minutes (considered as baseline, vertical dash line), and 6, 24, and 48 hours. (**A**, **B**) Proteostat spot size (*n*=8); (**C**, **D**) Proteostat spot intensity (*n*=8). (**E**) Representatives images of long-stored RBCs containing proteostat spots (i.e., orange dots) exposed to the physiological restoration model. Data are presented as mean ± SEM. Asterisks indicate significant differences within the DMSO group compared to the baseline (5 minutes), determined by two-way ANOVA followed by Dunnett's multiple comparisons test (* $p < 0.05$, ** $p < 0.01$, *** $p < 0.001$). Hash symbols indicate significant differences between the epoxomicin-treated and DMSO-treated groups at the same time point, determined by two-way ANOVA followed by Šídák's multiple comparisons test (non-significant). BF: Brightfield

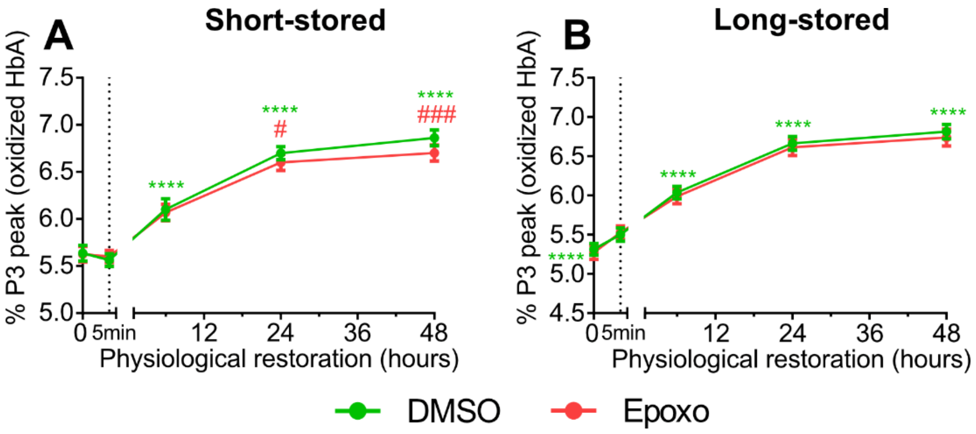

Supplemental Figure S6. Impact of proteasome inhibition on HPLC-P3 oxidized hemoglobin during physiological restoration of stored RBCs. RBC concentrates were treated with the proteasome inhibitor epoxomicin, (Epoxo, red circles), or vehicle control (DMSO, green circles) and then stored in SAGM solution at 4°C for 42 days. RBCs were exposed to a physiological restoration model on day 7 (short-stored, left panels) and on day 42 (long-stored, right panels). Samples were analyzed before physiological restoration and after 5 minutes (considered as baseline, vertical dash line), and 6, 24, and 48 hours. (**A**) Short-stored RBCs; (**B**) Long-stored RBCs. Data are presented as mean ± SEM (*n*=8). Asterisks indicate significant differences within the DMSO group compared to the baseline (5 minutes), determined by two-way ANOVA followed by Dunnett's multiple comparisons test (**** $p < 0.0001$). Hash symbols indicate significant differences between the epoxomicin-treated and DMSO-treated groups at the same time point, determined by two-way ANOVA followed by Šídák's multiple comparisons test (# $p < 0.05$, ### $p < 0.001$).

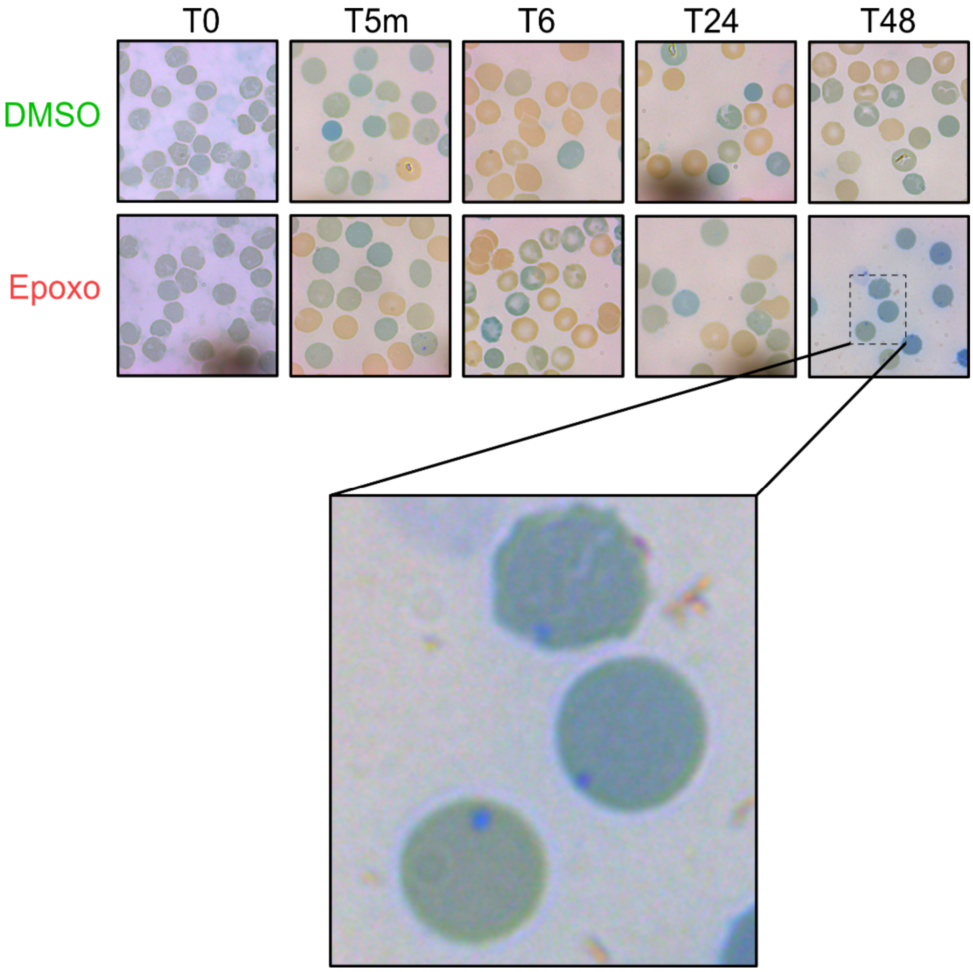

Supplemental Figure S7. Impact of proteasome inhibition on Heinz body production during physiological restoration of stored RBCs. RBC concentrates were treated with the proteasome inhibitor, epoxomicin (Epoxo), or vehicle control (DMSO) and then stored in SAGM solution at 4°C for 42 days. RBCs were exposed to a physiological restoration model on day 7 (short-stored, left panels) and on day 42 (long-stored, right panels). Samples were analyzed before physiological restoration, and after 5 minutes (considered as baseline), and 6, 24, and 48 hours. Representatives images of long-stored RBCs containing Heinz bodies (i.e., blue dots) which developed following exposure to the physiological restoration model.

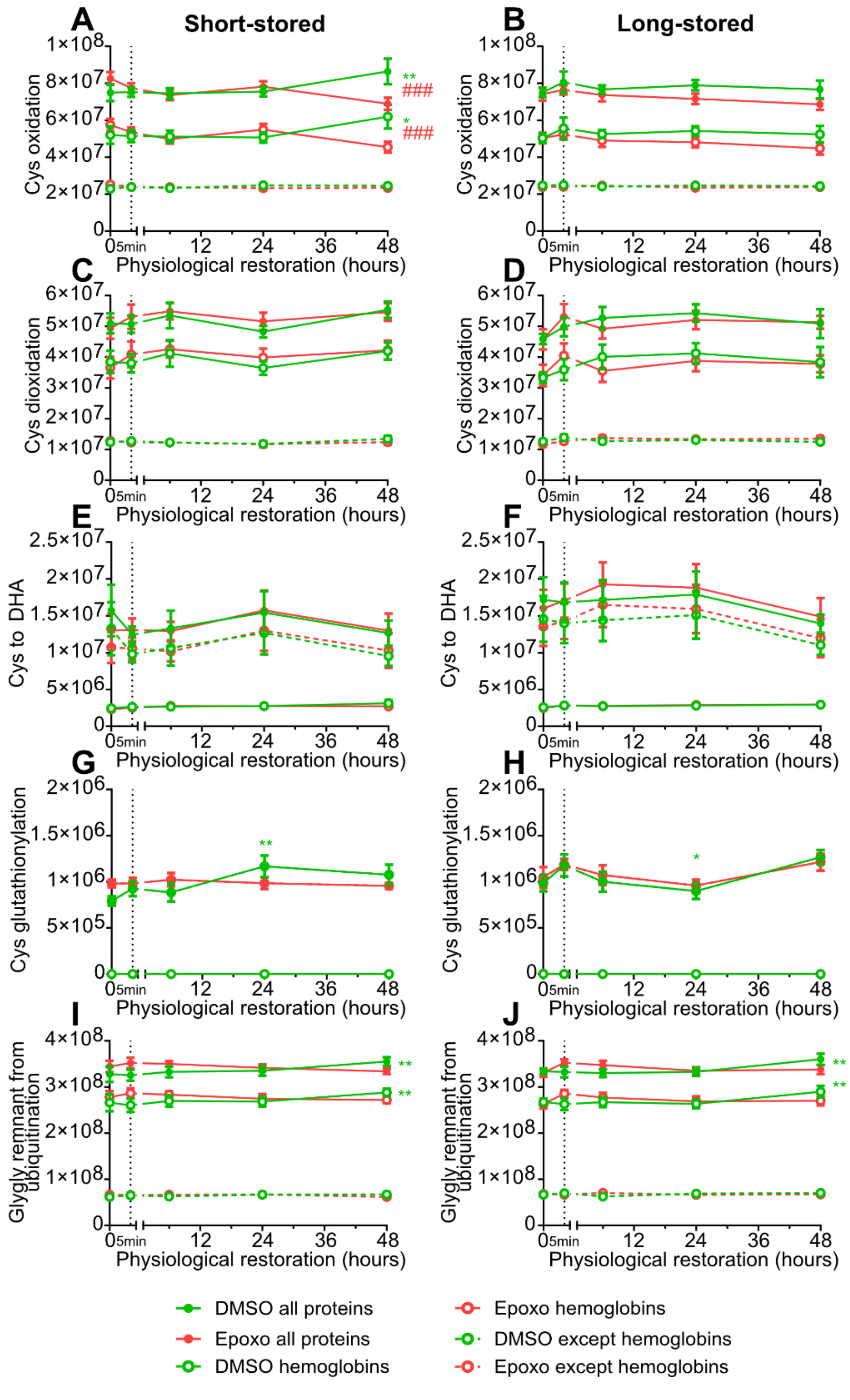

Supplemental Figure S8. Proteomic analysis of post-translational modifications during physiological restoration of stored RBCs. RBC concentrates were treated with the proteasome inhibitor, epoxomicin (Epoxo, red circles), or vehicle control (DMSO, green circles) and then stored in SAGM solution at 4°C for 42 days. RBCs were exposed to a physiological restoration model on day 7 (short-stored, left panels) and on day 42 (long-stored, right panels). Samples were analyzed by proteomics before physiological restoration and after 5 minutes (considered as baseline, vertical dash line), and 6, 24, and 48 hours. (**A**, **B**) Cysteine mono-oxidation; (**C**, **D**) Cysteine di-oxidation; (**E**, **F**) Cysteine converted to DHA; (**G**, **H**) Cysteine glutathionylation; (**I**, **J**) Gly-Gly remnants from ubiquitination. The

global analysis was obtained by summing intensities of all identified modified peptides. Proteins were divided into three groups: all proteins (solid circles with solid lines), hemoglobins (empty circles with solid lines), and all proteins except hemoglobins (empty circles with dashed lines). Data are presented as mean ± SEM (*n*=8). Asterisks indicate significant differences within the DMSO group compared to the baseline (5 minutes) (* p < 0.05, ** p < 0.01). Hash symbols indicate significant differences between the epoxomicin-treated and DMSO-treated groups at the same time point (### p < 0.001).

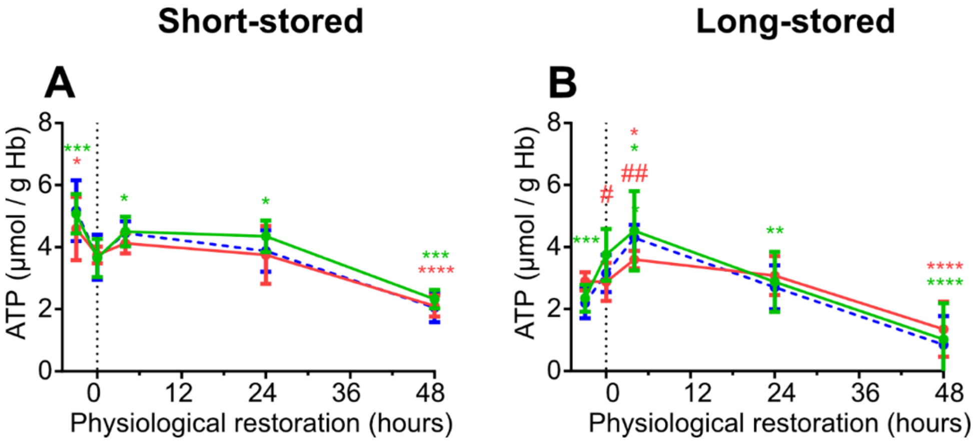

Supplemental Figure S9. Comparison of intracellular ATP level in young and old RBCs during physiological restoration. RBC concentrates were stored in SAGM solution at 4°C for 42 days. On days 6 and 42, young (green circles) *vs* old (red circles) RBC subpopulations were obtained by density gradient fractionation of samples from total RBC concentrates (blue circles). Subpopulations were exposed to a physiological restoration model on day 7 (short-stored, left panels) and on day 42 (long-stored, right panels). Samples were analyzed before physiological restoration and after 0 minutes (considered as baseline, vertical dash line), and 4, 24, and 48 hours. (**A**) Short-stored RBCs; (**B**) Long-stored RBCs. Data are presented as mean ± SD (*n*=4). Asterisks indicate significant differences within each group (green for young RBCs; red for old RBCs) compared to the baseline (0 minutes), determined by two-way ANOVA followed by Dunnett's multiple comparisons test (* p < 0.05, ** p < 0.01, *** p < 0.001, **** p < 0.0001). Hash symbols indicate significant differences between the young and old groups at the same time point, determined by two-way ANOVA followed by Šídák's multiple comparisons test (# p < 0.05, ## p < 0.01).
